# Cisplatin in Liver Cancer Therapy

**DOI:** 10.3390/ijms241310858

**Published:** 2023-06-29

**Authors:** Sae Hamaya, Kyoko Oura, Asahiro Morishita, Tsutomu Masaki

**Affiliations:** Department of Gastroenterology and Neurology, Kagawa University Faculty of Medicine, Kita-gun 761-0793, Japan; hamaya.sae@kagawa-u.ac.jp (S.H.); oura.kyoko@kagawa-u.ac.jp (K.O.); masaki.tsutomu@kagawa-u.ac.jp (T.M.)

**Keywords:** cisplatin, liver cancer, hepatocellular carcinoma, transarterial chemoembolization, hepatic arterial infusion chemotherapy, drug resistance

## Abstract

Hepatocellular carcinoma (HCC) is the most common primary liver tumor and is often diagnosed at an unresectable advanced stage. Systemic chemotherapy as well as transarterial chemoembolization (TACE) and hepatic arterial infusion chemotherapy (HAIC) are used to treat advanced HCC. TACE and HAIC have long been the standard of care for patients with unresectable HCC but are limited to the treatment of intrahepatic lesions. Systemic chemotherapy with doxorubicin or chemohormonal therapy with tamoxifen have also been considered, but neither has demonstrated survival benefits. In the treatment of unresectable advanced HCC, cisplatin is administered transhepatic arterially for local treatment. Subsequently, for cisplatin-refractory cases due to drug resistance, a shift to systemic therapy with a different mechanism of action is expected to produce new antitumor effects. Cisplatin is also used for the treatment of liver tumors other than HCC. This review summarizes the action and resistance mechanism of cisplatin and describes the treatment of the major hepatobiliary cancers for which cisplatin is used as an anticancer agent, with a focus on HCC.

## 1. Introduction

There are three major types of cancer treatment: surgical resection, radiation therapy, and drug therapy. Drug therapy is further classified into chemotherapy, molecular targeted therapy, and immunotherapy. Chemotherapy is used solely or in combination with surgical resection, radiation therapy, or other drug therapies, depending on the type and progression of the cancer [1,2,3].

The development of anticancer drugs began in 1946 when nitrogen mustard, used as a bioweapon during World War II, was found to have significant efficacy against malignant lymphomas [4]. Subsequently, nitrogen mustard was used as an alkylating agent in the treatment of malignant lymphoma and leukemia, and its derivative, cyclophosphamide, is used extensively [4]. Since then, new anticancer agents have continuously been developed and have drastically changed cancer treatment, which primarily constituted surgical resection and radiation therapy. Anticancer agents are broadly classified into antimetabolites, alkylating agents, anticancer antibiotics, microtubule polymerization and depolymerization inhibitors, topoisomerase inhibitors, and platinum agents, depending on their mechanism of action [1,2,3].

Cisplatin was the first platinum drug approved as an anticancer agent in the 1970s. Cisplatin and other platinum-based compounds have a broad spectrum of activity and are most effective in the treatment of various solid tumors, including testicular, ovarian, head and neck, bladder, lung, cervical, melanoma, and lymphoma, and are widely used to treat more than 80% of cancers [5,6].

Platinum agents exert anticancer effects through multiple mechanisms, the most common being induction of cell death by inhibiting DNA replication and transcription. However, as with other anticancer drugs, they damage normal cells along with the cancer cells; therefore, toxicity to organs other than the target organ (side effects) and the development of resistance are challenges that limit their use [6]. Although various similar compounds have been developed to reduce side effects, only two platinum drugs other than cisplatin, carboplatin and oxaliplatin, are currently certified worldwide and have been widely used in clinical practice since the initial development of cisplatin [7].

Systemic chemotherapy usually provides modest benefits in terms of disease control rates, progression-free survival, and overall survival, but at the cost of a substantial proportion of adverse events [8]. Hepatic arterial chemotherapy, even when combined with systemic therapy, has shown favorable results in terms of safety and efficacy as an equivalent or alternative to the gold standard therapy [8]. In addition, during the past few decades, new molecularly targeted agents have been developed and clinically evaluated as systemic chemotherapy, with interesting results. However, in many cases with poor prognosis and poor response to systemic therapy, specific inhibition of cancer cell signaling alone has proven to be insufficient [9]. Recently, it has become clear that one of the most important capabilities of tumors is the establishment of an immunosuppressive state within the tumor microenvironment. Within the liver, the hepatic microenvironment is generally oriented toward a state of immune tolerance, preventing autoimmune reactions [10]. The molecular biology of these events and the poor prognosis of advanced hepatocellular carcinoma is leading to the development of various immunotherapeutic strategies [10].

Hepatocellular carcinoma (HCC) is the most common primary liver tumor, with 70% to 80% of cases diagnosed at an unresectable advanced stage [11]. Therefore, transarterial chemoembolization (TACE) and systemic chemotherapy are essential to treat unresectable HCC; TACE has long been the standard of care for patients with unresectable HCC, but it is limited to the treatment of intrahepatic lesions. Systemic chemotherapy with doxorubicin or chemohormonal therapy with tamoxifen has also been investigated [12,13], but none of these chemotherapies have demonstrated a survival advantage. However, sorafenib is an effective systemic therapy for unresectable HCC in the 2007 SHARP trial [14], followed by lenvatinib in the REFLECT trial [15] and the combination of atezolizumab and bevacizumab in the IMbrave 150 trial [16]. The treatment of unresectable HCC is undergoing a major paradigm shift.

In the treatment of HCC, cisplatin is administered arterially for treatment; however, synergistic effects combined with systemic therapy have been anticipated in recent years.

Cisplatin is also used in the treatment of other liver tumors. Cisplatin-based chemotherapy is a common treatment for cholangiocarcinoma (CCA) and hepatoblastoma (HB).

This review summarizes the mechanism of action and resistance of cisplatin and describes the treatment of the major hepatobiliary cancers for which cisplatin is used as an anticancer agent, with a focus on HCC.

## 2. Development of Cisplatin

Cisplatin was synthesized by Michele Peyrone in 1845 and named the Peyrone salt, and its structure was deduced by Alfred Werner in 1893 [17,18,19]. However, its anticancer activity was not revealed until the 1960s, more than 100 years after its initial synthesis [17,18,19,20].

In 1965, Barnett Rosenberg et al. applied an electric current to a solution containing *E. coli* using a platinum electrode to study the effect of an electric field on the division of *E. coli* and found that the cell growth of *E. coli* was hindered [19,20]. Upon further research, they found that this phenomenon was not caused by an electric field but by a cis-diamminedichloroplatinum compound (i.e., cisplatin) that combined the platinum ions of the platinum electrode with ammonium chloride in the solvent [19,20]. In 1969, they examined the administration of cisplatin to mammalian systems and tested its effect on inhibiting the cell division of sarcoma 180 and leukemia L1210 cells in a mouse model. Their study results showed that it suppressed rat sarcoma and prolonged the mean survival period [21,22].

Based on these results, clinical trials initiated in 1971 confirmed its efficacy as an anticancer drug, particularly in testicular and ovarian cancer; however, its strong toxicity, including nephrotoxicity, ototoxicity, and neurotoxicity, was concerning [18,19,23]. However, after nearly a decade, the US Food and Drug Administration (US FDA) approved it for use against ovarian and testicular cancer for the first time in 1978, partly because renal damage could be reduced by large fluid load and diuretic use [18,19,23]. Since then, cancer treatment options have increased significantly.

## 3. Mechanism of Cisplatin

### 3.1. Intracellular Transport of Cisplatin

Cisplatin is generally used in vivo by intravascular administration. Following intravenous injection in humans, 68–98% of cisplatin in the blood is bound to proteins (particularly albumin) via histidine (His) and methionine (Met) residues [24,25,26]. The mechanism underlying cisplatin uptake into cells is not entirely clear; cisplatin enters the cell primarily through passive mechanisms such as diffusion [26,27,28,29]. This has been widely shown in vitro [27,28,29,30] and is proportional to the extracellular concentration up to a concentration of 3 mM [26].

**Figure 1:**
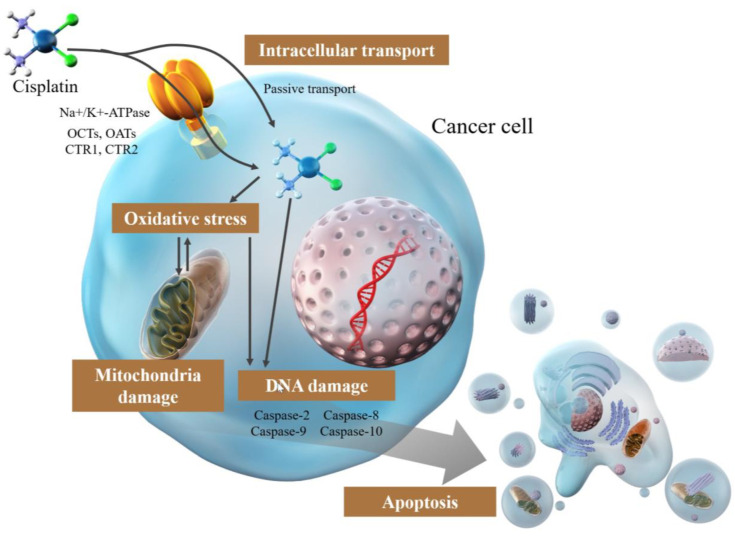
Mechanism of the effect of cisplatin in cancer cells.

Conversely, the involvement of active transport systems [26,27,28,29,31,32,33,34], including Na^+^/K^+^-ATPase [27,29] and organic cation and organic anion transporters (OCTs and OATs) that are highly expressed in tissues sensitive to cisplatin toxicity, such as the kidney, cochlea, and auditory nerve [26,27,31,32], has been proposed, but no particular inference has been made (Figure 1). Another hypothesis is copper transporters [27,28,33,34,35,36], SLC31A1 and SLC31A2, which have numerous His and Met residues, indicating that they may bind cisplatin by mimicking binding to albumin binding [26]. CTR1, a member of the SLC31A1 family, is involved in cisplatin uptake and tolerance in experiments using yeast and mice where CTR1 was knocked out [34], and cisplatin-resistant cell lines were reported to have lower intracellular copper levels overall [37], indicating a correlation between copper transporters and cisplatin uptake (Figure 1). However, some of the pathways are skeptical: the binding of SLC31A1 (CTR1) and SLC31A2 (CTR2) to Met residues does not alter the rate of cisplatin entry in terms of stability or following an increase in CTR1 expression. The failure of cisplatin binding to 31A2 (CTR2) and Met residues may imply uncertainty or non-involvement of these residues in the cellular uptake of cisplatin [26,27,38,39]. Therefore, numerous aspects of active transport of cisplatin remain to be elucidated. Moreover, the transport mechanisms involved, and the extent of this involvement are unclear. Further studies are needed to elucidate the mechanism underlying cisplatin uptake, which is essential to elucidating the resistance mechanism.

### 3.2. Damage via DNA Cross-Linking by Cisplatin

The main target of cisplatin is DNA, which exerts anticancer effects by forming covalent adducts with DNA and causing damage.

Regarding the structure of cisplatin, two chloride atoms and two ammonia molecules are bound to the central metal, platinum (II) atom, in the cis position [17]. The chloride atoms are highly unstable and reversible under certain pH and temperature conditions and can be substituted, while the bond between the platinum and ammonia molecules is irreversible and substitution inert [17,19,20]. When cisplatin is taken up into the cell from the blood, the chloride atoms are displaced by water molecules. Cisplatin is relatively unreactive and stable in blood with high chloride concentrations (100 mM) because the substitution of the chloride ligand is prevented [18,27,40]. However, in cells with low chloride concentrations (4 mM), the chloride ligand is hydrolyzed and replaced by a water molecule [18,40]. Hydrolyzed cisplatin is highly reactive and serves as a potent electrophile reacting with nucleophiles such as nitrogen atoms of nucleic acids and SH groups of proteins, which are variously covalently bound to DNA, forming covalent DNA adducts [19,40,41]. Platinum atoms bind preferentially to the nitrogen atom at position 7 of the guanine and adenine bases in particular, bridging two adjacent purine bases [19,40,41]. Most of the cross-links formed by these adducts (approximately 90%) are the 1,2-intrastrand cross-links (1,2-intrastrand (GpG) and 1,2-intrastrand (ApG)); a smaller proportion of interstrand cross-links and monofunctional adducts are also formed [19,40,42,43]. These DNA adducts inhibit the DNA replication machinery and affect transcription [19,41,44].

### 3.3. Cisplatin-Induced DNA Replication and Transcription Arrest

Cancer cells that are damaged by DNA damage, owing to the formation of DNA adducts, can have their DNA replication inhibited and their cell cycle arrested at the G1, S, and G2/M phases [45,46]. Distortion of DNA strands by cross-linking affects RNA transcription arrest [46]. Although the mechanism of transcriptional regulation has not been fully elucidated, mechanisms such as polymerase and transcription factor inhibition [47,48,49,50] and disruption of chromatin structure by the formation of DNA adducts have been considered [50,51,52,53].

RNA polymerase II (Pol II) is an essential enzyme for DNA transcription, and transcription by RNA polymerase II requires six basic transcription factors: TFIIA, TFIIB, TFIID, TFIIE, TFIIF, and TFIIH. TFIID is particularly important because it contains a TATA-binding protein (TBP) that binds to the TATA box, a sequence that defines the start position of transcription. Transcription is initiated from the correct position. However, TBP binds directly to the TATA box and the cisplatin-DNA adduct. Consequently, this adduct may prevent transcription initiation by RNA polymerase II by blocking the binding of TBP to the TATA box [47]. This inhibition of transcription by the DNA adduct hijacking mechanism has also been observed for the human upstream binding factor (hUBF), ribosomal RNA (rRNA) transcription factor [48,49].

DNA is wrapped around eight histone proteins to form a structure called a nucleosome, and the structure of many of these is called chromatin. Chromatin remodeling plays an important role in the transcription machinery because the tight wrapping of DNA around histone proteins is a major obstacle for the transcription machinery to access DNA. Chromatin remodeling allows transcription-related proteins, such as RNA polymerase, to access DNA by loosening chromatin aggregation through covalent histone modifications and by moving nucleosomes in an ATP-dependent manner. Cisplatin inhibits chromatin remodeling in vitro and in vivo [50] and intrastrand and interstrand cross-linking is involved in this inhibition of chromatin remodeling [51,52,53]. However, there are numerous reports on the mechanism of cisplatin-induced DNA damage-induced transcriptional inhibition, which partially explain the mechanism.

### 3.4. Induction of Apoptosis by Cisplatin

Apoptosis is induced by the activation of caspases via exogenous or endogenous cell death signals. In the exogenous pathway, caspase-8 is activated by the binding of TNF or FasL to the cell surface cell death receptors, TNF receptor (TNFR), or Fas. Conversely, endogenous pathways are activated by factors such as cellular stress and DNA damage and are mediated by mitochondria. These are regulated by Bcl-2 family proteins, including those that promote apoptosis, such as Bax and Bak, and those that inhibit apoptosis, such as Bcl-2 and Bcl-xL. These alter the permeability of the mitochondrial outer membrane, release cytochrome c into the cytoplasm, and activate Caspase-9 by interacting with Apaf-1. Initiator caspases, such as caspase-2, -8, -9, and -10, are activated, followed by the activation of executive caspase-3, -7, and apoptosis. Cisplatin induces apoptosis through protein kinase C (PKC), mitogen-active protein kinase (MAPK), Jun-amino-terminal kinase (JNK), p53, and Akt, which are molecules that constitute signaling pathways that stimulate these cascades.

Signaling pathways that stimulate these cascades include PKC, MAPK, JNK, p53, and Akt [45]. PKCs are a family of lipid-dependent serine-threonine kinases, which are also classified into three groups: conventional (α, β1, βII, γ), novel (δ, ε, η, θ), and atypical (ζ, β1, βII, γ). PKCδ is a positive regulator of cisplatin-induced cell death; for human gastric cancer cells MKN28 with mutations in the p53 gene, PKCδ may cooperate with p53 to regulate caspase-3-mediated cell death [46]. Cisplatin-induced DNA damage activates extracellular signal-related kinase (ERK) via PKCδ. Three subfamilies of the MAPK family have been identified: the ERK, JNK, and p38 kinases [47]. Among them, ERK phosphorylates p53 and activates p53 [48]. Activated p53 upregulates p21, 45kd-growth arrest and DNA damage (GADD45), and mouse double minute 2 homolog (Mdm2) [49], causing cell cycle arrest and allowing time for DNA repair [50]. If damage is not repaired, p53 activates transcription of Bax and represses transcription of Bcl-2, directly affecting the expression of downstream genes that regulate apoptosis susceptibility [51].

DNA damage activates JNK, which is observed in both cis and transformation of cisplatin. p73 forms a complex with JNK and induces cisplatin-induced apoptosis [53]. p73, similar to p53, also upregulates the expression of apoptosis promoting proteins such as Bax and PUMA, and initiates apoptosis by upregulating their expression [50]. Furthermore, the JNK signaling pathway may induce exogenous apoptosis involving FasL via p38 [51]. In addition, DNA damage mobilizes the tyrosine kinase receptor c-Abl from the cytoplasm to the nucleus, where it binds and phosphorylates MEK kinase 1, a complex that activates jun amino-terminal kinase/stress-activated protein kinases (JNK/SAPK). However, activation by c-Abl is conditional upon recognition of DNA damage by the mismatch repair (MMR) system, and MMR-deficient cells do not respond to c-Abl [19].

Through the MAPK cascade, p38 induces stabilization of p18 (Hamlet), which in turn induces apoptosis through the interaction of p53 with apoptosis-promoting genes, PUMA and NOXA [54]. Other possible mechanisms include degradation of flice-like inhibitory protein (FLIP), direct binding of B-cell lymphoma-extra-large (Bcl -xL) and counteracting of its anti-apoptotic function, phosphatase and tensin homolog (PTEN) overexpression, and inhibition of AMPK [19].

### 3.5. Mitochondrial Damage by Cisplatin

DNA is present in the mitochondria and the nucleus, making them a target of cisplatin-induced DNA damage and causing mitochondrial damage [18,55,56]. Mitochondria produce ATP through the electron transport system, generate reactive oxygen species, and are important mediators of apoptosis; therefore, cisplatin-induced mitochondrial damage induces apoptosis [57]. Cisplatin directly damages mitochondrial DNA, resulting in increased intracellular reactive oxygen species (ROS) levels from decreased mitochondrial protein synthesis and impaired electron transfer system function [12,58]. The production of ROS is highly dependent on mitochondrial damage [18,58]. The generated ROS attack the mitochondria and produce additional ROS.

Cells maintain ROS levels in equilibrium by balancing ROS production and scavenging systems [57,59]. Scavenging systems include enzymatic scavengers such as superoxide dismutase (SOD) and catalase, and non-enzymatic scavengers such as glutathione (GSH) [57]. Moderate amounts of ROS are necessary for tumor promotion, but excessive ROS suppresses the tumor [59]. Therefore, when ROS production overwhelms the scavenging system, excess ROS induces apoptosis via endogenous or exogenous pathways [19,57,58,59].

In the extrinsic pathway, ROS activates death-causing receptors on the plasma membrane, such as Fas, TRAIL-R1/2, and TNF-R1 [19,57,59]. In the endogenous pathway, ROS promotes cytochrome c release via activation of p53 and JNK, depolarization of mitochondrial membranes, and Bax and Bak [5,11]. Furthermore, depending on the amount of ROS generated, they may bring about necrosis and autophagy as well as apoptosis in cancer cells [60,61].

## 4. Mechanisms of Cisplatin Resistance

The development of drug resistance, as well as side effects, is one of the major hindrances to treatment continuity in cancer chemotherapy. The effective concentration of cisplatin in cancer therapy is limited to a fairly narrow range and increasing the dosage may ultimately increase side effects. This complicates the prevention of cisplatin resistance by increasing therapeutic doses [22].

**Figure 2:**
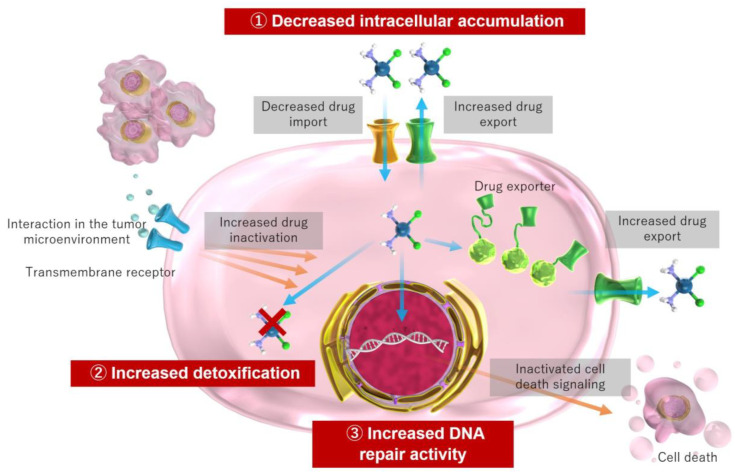
Mechanism of cisplatin resistance in cancer cells.

The acquisition of resistance occurs through various mechanisms at the molecular and cellular levels. These mechanisms include decreased intracellular accumulation of cisplatin via intracellular drug influx and efflux, increased detoxification through increased intracellular thiol levels via glutathione and metallothionein, increased DNA repair activity, and prevention of apoptosis via signaling molecules among other mechanisms [18,22,41,45,62] (Figure 2).

Decreased intracellular accumulation of cisplatin is primarily associated with decreased uptake and markedly increased efflux [41]. The copper transport protein, CTR1, may be involved in the cellular uptake of cisplatin, and deletion of the CTR1 gene results in increased cisplatin resistance and decreased intracellular accumulation of cisplatin [34]. In cisplatin-resistant cell lines, CTR1 expression and intracellular accumulation of cisplatin is decreased [18,63,64].

Conversely, transporter proteins ATP7A and ATP7B, and the multidrug resistance-related protein MRP2, have been implicated in increased efflux [41,64,65]. Cisplatin binds to the antioxidant glutathione to form glutathione conjugates, which are then exported via MRP2 [66,67]; increased MRP2 may be involved in cisplatin resistance [26,65,68]. Therefore, the response that hinders cisplatin retention in cancer cells is a major form of acquired resistance.

In addition to facilitating efflux, glutathione is involved in detoxification and ROS removal [64,68]. Glutathione and metallothionein reduce toxicity by coordinating cisplatin to thiol groups [41,66,68]. Therefore, elevated glutathione levels may be involved in cisplatin resistance, and elevated glutathione levels have been observed in certain cisplatin-resistant cells [69,70].

DNA damage repair mechanisms may also be involved in the resistance mechanism. DNA repair mechanisms are enhanced in cisplatin-resistant cells and suppressed in cisplatin-sensitive cells [71]. DNA repair mechanisms that work against cisplatin-induced DNA damage include nucleotide excision repair (NER), homologous recombination (HR), and mismatch repair (MMR) [41].

Intrastrand cross-links are mainly repaired by NER, and ERCC1 (excision repair cross-complementing 1) and XPF (xeroderma pigmentosum complementation group F) are involved in this repair pathway. These proteins form a protein heterodimer, cleaving the 5′ end of the strand where cisplatin and DNA bind, allowing for the removal of the adduct [41]. The relationship between increased expression of ERCC1 and XPF and cisplatin resistance have been reported in past studies [16,17,18]. In addition, the expression of replication protein A (RPA) 14 [72] and xeroderma pigmentosum group C (XPC) [73], which are involved in NER, are associated with the expression of mi-RNA 488. miR-488 activates NER by suppressing eIF3a expression and increasing XPC and RPA 14 expression, thus contributing to cisplatin resistance [74].

Intrastrand cross-links are repaired by HR, a repair mechanism for double-strand breaks (DSBs), which involves BRCA-1 and BRCA-2 [41,75]. Mutations in these genes may contribute to cisplatin sensitivity or resistance [76,77]. RAD21 is involved in HR, and low expression of miR-17 and miR-92 families may promote DNA damage repair by increasing RAD21 expression and contribute to cisplatin resistance [78].

The loss of MMR function may also be associated with increased resistance; MSH2 and MLH1 genes involved in MMR are mutated or downregulated by cisplatin resistance, and apoptosis is suppressed [41]. In addition, the aforementioned apoptotic mechanisms involving c-Abl and JNK are absent in MMR-deficient cells [19,79].

One resistance mechanism that interferes with cell death by DNA adducts is inactivation of the T53 gene encoding p53, which results in the loss of apoptotic control and the development of resistance in 50% of human cancers [80]. In addition, there are various cisplatin resistance mechanisms, including changes in various proteins involved in apoptosis-inducing signaling, inhibition of antioxidant enzyme activity and autophagy, and involvement of miRNAs [18,22,41,45,62]. The acquisition of resistance clearly poses significant clinical problems, and further research is needed to address this problem.

## 5. Adverse Events of Cisplatin

Despite being an effective cancer treatment, cisplatin is toxic to normal organs. The mechanism and extent of toxicity to normal cells, as well as prophylactic and therapeutic measures, are largely unknown. Despite the fact that toxicity is dose-dependent and overdosage can have severe consequences, related incidence is unknown and guidelines for overdosage have not been established [45]. Cisplatin has various side effects, including general cytotoxic effects such as nausea and vomiting, decreased blood cell production in the bone marrow (myelosuppression), immunosuppression, hepatotoxicity, and cardiotoxicity (Table 1). In particular, the kidneys, auditory organs, and peripheral nerves are the most commonly affected organs, and nephrotoxicity, ototoxicity, and neurotoxicity are typical and severe side effects (Table 1) [18,22,45,81,82].

In clinical practice, cisplatin treatment-induced acute kidney injury occurs in one-third of treated patients approximately 10 days after treatment [81,83,84,85]. Nephrotoxicity is a common limitation of cisplatin. Cisplatin is primarily excreted via the glomerular filtration and tubular functions of the kidney [86]. Cisplatin accumulates in nephron proximal tubular epithelial cells at more than five times the serum concentration [22,84], and is contained in higher levels in the kidney than in any other tissue. As mentioned earlier, CTR1 and OCT2 are involved in the cellular uptake of cisplatin; CTR1 is highly expressed in proximal and distal renal tubular cells and is localized to the basolateral tubules, the main site of cisplatin uptake [87]. In vitro studies have shown that OCT2 is also highly expressed in the proximal tubular basement membrane, and the cellular accumulation and nephrotoxicity of cisplatin is enhanced by OCT2 expression [31,88]. The loss of OCT2 decreases urinary excretion and nephrotoxicity of cisplatin [89].

Cisplatin accumulated in renal cells undergoes metabolic activation in the kidney to become a more potent toxin and damages nuclear and mitochondrial DNA, causing ROS generation and activating endogenous and exogenous apoptosis [90]. Although ROS [91,92] and apoptotic [93,94] activity contribute to renal cell death, many of these pathways also contribute to the antitumor mechanism of cisplatin. Therefore, strategies to reduce renal injury may result in a reduction of the antitumor effect of cisplatin and must be carefully considered [90]. Inflammatory mechanisms have also been implicated in the pathogenesis of cisplatin nephrotoxicity [95,96,97,98]. The injury of renal epithelial cells by cisplatin results in the production of various inflammatory cytokines and chemokines; the activations of TNFα [95,96,97] and T lymphocytes [98] are among these inflammatory mechanisms.

Hydration, mannitol, and magnesium effectively prevent cisplatin-induced nephrotoxicity [83]. Hydration significantly decreases proximal tubular transit time and significantly decreases the half-life of cisplatin and urinary cisplatin concentration [83]. Short-term, low-dose hydration can be performed on an outpatient basis, allowing for relatively safe continuation of cisplatin therapy without compromising cisplatin’s anticancer effects [99,100,101,102].

Magnesium supplementation with hydration inhibits the nephrotoxicity of cisplatin [103,104,105,106]. Magnesium may be involved in transporters for cisplatin intracellular influx or excretion, and magnesium deficiency increases intracellular accumulation [107,108,109]. This phenomenon may be more pronounced in normal renal cells than in cancer cells, and magnesium supplementation may play a renoprotective role without compromising the anticancer effects of cisplatin [108]. In addition, the concomitant use of mannitol, an osmotic diuretic, is beneficial in the prevention of nephrotoxicity when high-dose cisplatin is administered [110,111], although some reports point to the possibility of hyponatremia [112]. Moreover, there is no proof that the use of mannitol is more nephroprotective than that of hydration alone, and further studies are needed [113,114].

Ototoxicity has a high incidence rate, with 20–70% of patients experiencing cisplatin-related ototoxicity and 40–80% developing permanent hearing loss [18]. This causes progressive, irreversible, bilateral high-frequency hearing loss, tinnitus, and ear pain [115]. CTR1 is expressed in outer and inner hair cells, choroid and spiral ganglia, and OCT2 in the Corti organ and choroid [116]. Similar to nephrotoxicity, ototoxicity also occurs when cisplatin accumulates in cochlear cells, causing DNA damage, generating ROS, and inducing inflammation and apoptosis [116]. Therefore, even for ototoxicity, it is important to reduce toxicity without decreasing anticancer activity. Although no treatment or prophylaxis has been established, local treatment with intratympanic administration of otoprotective agents may a novel way to prevent systemic effects [116].

Neurotoxicity is another side effect, with frequency of occurrence ranging from 19% to over 85% [117]. Cisplatin accumulation primarily damages the dorsal root ganglia and manifests as peripheral sensory neuropathy characterized primarily by “stocking and glove” distribution of paresthesias, paresthesias, and numbness [118,119,120]. Abnormal sensations, such as burning, pain, and decreased sensitivity to vibration, often persist or increase for months or longer after treatment ends, and these long-term effects are associated with depression, insomnia, falls, and reduced health-related quality of life [121,122]. Similar to nephrotoxicity and ototoxicity, the mechanism of neurotoxicity involves mitochondrial damage and induction of apoptosis via ROS. Cisplatin also activates glial cells, immune cells, and inflammatory cytokines, hyper-excites peripheral nerve cells by activating Na+, K+, and TRP ion channels, and damages the blood–brain barrier [117,122]. No effective treatment has been established, and the efficacy of α-lipoic acid, glutathione, amifostine, and vitamin E have been investigated, but data to support clinical application are not yet available [123].

Although cisplatin is a very useful anticancer agent, these toxicities limit its usefulness, and elucidating and controlling the mechanisms of this toxicity may be critical in successful cancer chemotherapy.

**Table 1:** Incidence of each adverse event by cisplatin for HCC.

nausea	44%	[124]
vomiting	44%	[124]
myelosuppression	32%	[125]
immunosuppression	18%	[125]
hepatotoxicity	18%	[126]
cardiotoxicity	6%	[127]
nephrotoxicity	30–40%	[84,85,86,90]
ototoxicity	20–70%	[116]
neurotoxicity	19% to over 85%	[117,118,119,120]

## 6. Cisplatin for HCC Treatment

### 6.1. Epidemiology and Treatment Algorithm for HCC

Primary liver cancer is the sixth most commonly diagnosed cancer worldwide with approximately 906,000 new cases, and is the third leading cause of cancer death with approximately 830,000 deaths in 2020 [128,129]. HCC is common in East Asia, Southeast Asia, and North and West Africa, with increasing incidence in Europe and the United States [128,130]. HCC accounts for 75–85% of primary liver cancers, followed by intrahepatic cholangiocarcinoma, which accounts for 10–15% of these cancers [128]. Major risk factors vary according to region, with hepatitis B virus infection in Asia, hepatitis C virus infection in Japan, and alcoholic liver disease and non-alcoholic fatty liver disease in Europe and North America [129]. The survey mechanism of these patients for early detection of HCC is important [131].

The Barcelona Clinic Liver Cancer (BCLC) staging system is widely used to classify patients into very early (0), early (A), intermediate (B), advanced (C), and terminal (D) stages according to the progression stage and the patient’s performance status. Trans-arterial chemoembolization (TACE) is a local treatment method for inducing tumor necrosis by the anticancer effect of local retention of anticancer drugs and the inhibitory effect of embolization of tumor nutrient blood vessels, and cisplatin, doxorubicin, and epirubicin are often used, with cisplatin being the most commonly used for HCC [132]. Despite numerous reports showing the usefulness of cisplatin in TACE for HCC [133,134,135,136], no inference on the superiority of any particular drug has been made [137]. According to the European Association for the Study of the Liver (EASL) guidelines in 2018, TACE was mainly the first choice in the intermediate stage [2]. However, with the advent of molecular targeted agents (MTAs) and immune checkpoint inhibitors (ICIs), MTAs and/or ICIs have become more effective even in the intermediate stage [138,139]. Therefore, according to the 2022 update, TACE is recommended for HCC in the intermediate stage with well-defined tumor size, and selective embolization with preserved portal vein blood flow and systemic chemotherapy are recommended for diffuse, invasive, and extensive lesions [140]. The concept of treatment stage migration (TSM), where a change to a preferred therapy at a different stage is recommended if the initial recommendation fails or depending on the individual patient profile, has been incorporated [140]. TACE may be recommended even at very early-stage HCC.

Another HCC treatment using cisplatin is hepatic arterial infusion chemotherapy (HAIC), which delivers high concentrations of anticancer drugs directly to target sites in the liver via the hepatic artery, allowing for stable and continuous local administration of anticancer drugs, thereby keeping the systemic concentration of anticancer drugs low. Although the efficacy of HAIC using a regimen of cisplatin plus 5-FU has been reported [141,142,143], HAIC is generally not recommended owing to the lack of evidence of improved prognosis in large randomized trials of HAIC in patients with advanced HCC, and the risk of vascular injury due to catheter and reservoir placement and management [144].

### 6.2. Molecular Mechanisms in TACE

As mentioned above, TACE is administered by injecting chemotherapeutic agents directly into the hepatic artery that feeds the tumor and embolizing the artery with an embolic substance. The ischemia caused by embolization leads to hypoxia in hepatocytes and surrounding liver tissue [145]. In HCC, the hypoxia inducible factor (HIF) signaling pathway is stimulated under hypoxia induced by TACE, among which, HIF1α is expressed in an oxygen-dependent manner [146]. The induction of HIF-1α by hypoxia promotes the expression of VEGFA, FGF2, and PDGFA in HCCs; VEGFA binds to VEGFR2 and activates the PI3K/ACT and RAF/MAPK pathways, whereas FGF2 interacts with fibroblast growth factor receptor 1 (FGFR1) and activates the RAF/MAPK pathway. By activating the RAF/MAPK pathway, PDGFA activates MEK/ERK signaling via platelet-derived growth factor receptor (PDGFR) [86]. These mechanisms promote HCC angiogenesis. The newly formed vessels are usually hyperpermeable, creating areas of high interstitial pressure and severe hypoxia or necrosis, promoting HCC progression and angiogenesis [86]. The decrease in HIF1α expression in endothelial cells of tumor vessels reduces the expression of VEGF and suppresses vascular growth and tumor size [147]. Serum VEGF in HCC patients after TACE administration has been found to increase significantly with prolonged high levels associated with distant metastasis and tumor growth [145,148,149]. Poor therapeutic effects of TACE are associated with tumor angiogenesis in residual lesions after TACE, and HIF1α and VEGF may play an important role in the regulation of these angiogenesis [150].

Regarding epigenetic changes after TACE, decreased miRNA-125b is involved in the recurrence of HCC patients after adjuvant TACE [151]. miR-125b inhibits the translation of HIF1α in HCC and is an important tumor suppressor [151]. This indicates that decreased miRNA-125b contributes to HIF1α activation and may be involved with resistance to TACE [151]. Furthermore, miR-200a [121], miR-133b, miR-26a, miR-107, and miR-106 [152], and exosomal miR-122 [153] are miRNA changes that can be utilized as prognostic biomarkers for TACE, but further studies are needed to determine differences among different anticancer agents including cisplatin.

In addition, circ-G004213 in exosomes is a potential predictor of the efficacy of TACE with cisplatin [154]. Exosomes contain active substances such as DNA and noncoding RNA (ncRNA), and circular RNA (circRNA), a type of ncRNA, is enriched in exosomes and plays an important role in cancer biology [155]. Many circRNAs act as miRNA sponge molecules and regulate gene expression [155]. High expression of circ-G004213 by TACE interacts with miR-513b-5p and its target gene PRPF39, upregulating PRPF39 after TACE and RPF39 was associated with cisplatin sensitivity [m], indicating that circ-G004213/miR-513b-5p/PRPF39 may be effective for predicting the therapeutic effects of cisplatin-based TACE [154].

### 6.3. Systemic Therapy and Cisplatin-Based TACE for HCC

Recently, the combination of TACE with systemic therapy for their cumulative effect has been investigated. Six chemicals have been approved as systemic therapies for unresectable HCC based on Phase III clinical trials: sorafenib [156], regorafenib [157], lenvatinib [15], cabozantinib [158], ramucirumab [159], and atezolizumab plus bevacizumab (atezo + bev) [16].

Systemic therapy for HCC is dominated by the combination of MTAs sorafenib and lenvatinib and ICIs atezolizumab and bevacizumab, and the combination of these agents with combination therapy with TACE is considered to be a new and potentially effective strategy. The combination of TACE with sorafenib improves progression free survival (PFS) and significantly prolong overall survival (OS) [160]. In addition, lenvatinib-TACE sequential therapy, where TACE is performed after prior administration of lenvatinib, is effective [139,161,162]. The effects of prior administration of lenvatinib are synergistic effects: (1) tumor shrinkage and necrosis, (2) curative TACE and preservation of hepatic functional reserve, (3) normalization of blood vessels for efficient and uniform distribution of anticancer drugs, and (4) inhibition of angiogenesis induced by hypoxia after TACE through VEGF inhabitation [65].

Tyrosine kinases inhibited by MTAs are a variety of proteins, including RTKs, which are phosphorylated to regulate cell growth, differentiation, and death through various intracellular signaling molecules (Table 2) [9]. RTKs typically involved in tumorigenesis include VEGFR, EGFR, FGFR, PDGFR, and insulin receptors (INsR) [9]. The target molecules of each of the MTAs are listed in Table 1. The combination of TACE and MTAs not only provides anti-proliferative and anti-angiogenic effects of these molecules via these molecules, but also reduces vascular permeability and tumor stromal pressure by normalizing blood vessels, which may improve the intratumor distribution of anticancer drugs [162,163].

**Table 2:** Molecular targeted agents and immune checkpoint inhibitors for HCC.

	Drug	Target	References
Tyrosine Kinase Inhibitors	Sorafenib	Almost 40 tyrosine kinases, such as c-RAF, B-RAF, VEGFR1-3, PDGFR-α/β, c-Kit, FLT-3, and RET	[9,156,164]
	Regorafenib	c-RAF, wild-type and mutant (V600E) B-RAF, VEGFR1-3, FGFR1-2, PDGFR, KIT, RET, angiopoietin 1 receptor (TIE2), and p-38-α (greater potency to target VEGFR, KIT, TIE2, and RET compared to Sorafenib)	[9,164,165]
	Lenvatinib	VEGFR1–3, FGFR1-4, PDGFR-α, KIT, and RET	[9,15,164]
	Cabozantinib	VEGFR 1–3, KIT, RET, TIE2, FLT3, c-MET, and AXL	[9,158,164]
VEGF Inhibitors	Ramucirumab	VEGFR-2	[9,159,164]
	Bevacizumab	VEGFR2 by binding VEGF-A	[9,164,165]
Immune Checkpoint Inhibitor	Atezolizumab	PD-L1	[9,164,165]

c-RAF, c-rapidly accelerated fibrosarcoma; B-RAF, b-rapidly accelerated fibrosarcoma; VEGFR, vascular endothelial growth factor receptor; PDGFR-α/β, platelet-derived growth factor receptor-α/β; FLT-3, fms like tyrosine kinase 3; RET, rearranged during transfection; FGFR1-4, fibroblast growth factor receptors 1-4; TIE2, tyrosine kinase with immunoglobulin-like and EGF-like domains; VEGFA, vascular endothelial growth factor A; PD-L1, programmed death-ligand 1.

There is also growing evidence regarding the efficacy of HAIC combined with systemic therapy for HCC. Recently, a randomized clinical trial of sorafenib combined with cisplatin-based HAIC was conducted, and in a randomized phase 2 trial comparing HAIC plus sorafenib versus sorafenib alone, the primary endpoint of OS was superior in the HAIC plus sorafenib [17]. In addition, a phase 3 study (SILIUS study) comparing the combination of sorafenib and HAIC with cisplatin plus 5-FU to sorafenib alone did not achieve a prolonged OS benefit with the combination of sorafenib and HAIC, but a sub-analysis showed that only the tumor plug in the advanced portal vein had an additive effect [166].

Notably, TACE or HAIC with cisplatin may also be effective in the treatment of MTA-resistant HCC, which is a clinical problem. In a basic study, we reported that cisplatin induces G2/M cell cycle arrest through DNA damage response via the ATM/ATR-Chk1/Chk2 signaling pathway in lenvatinib-resistant HCC and shows antitumor effects [167], and that in a real clinical setting, cisplatin combination on-demand TACE was effective in patients with intrahepatic metastases who were resistant to sorafenib and lenvatinib, leading to re-administration of lenvatinib [168]. Other authors have reported cases demonstrating the efficacy of drug-eluting beads (DEB)-TACE with cisplatin for rapidly growing tumors after atezolizumab plus bevacizumab therapy [169] and HAIC for HCC with an inadequate response to initial TACE treatment and lenvatinib [170].

Regarding immunotherapy, in tumor specimens from surgically treated HCC patients, the effects of TACE on the immune system have become clear, including that expression of programmed death-1 (PD-1) and programmed death-ligand 1 (PD-L1) in tumor (Table 2) is considerably higher in patients undergoing preoperative TACE [171], TACE-induced tumor cell necrosis increases the release of tumor-associated antigens and CD4+ T cells [172]. Cisplatin also enhances PD-L1 expression in certain cancer types and may be effective in combination therapy with ICI [173,174], and a similar mechanism has been suggested in HCC [175,176]. These findings suggest that combination therapy with TACE and ICI using cisplatin may be a promising treatment option. In addition, clinical trials of the three-drug combination of TACE, MTAs, and ICI are underway, with the three-drug combination achieving a conversion rate to surgical resection as high as 42%, indicating that surgical resection can maximize the outcome of patients with unresectable HCC [177].

With the rapid spread of clinical use of MTA and ICI, synergistic effects can be expected from the combination of systemic therapy and cisplatin-based TACE or HAIC, and this combination therapy is expected to be a new treatment strategy for MTA- and ICI-resistant HCC that is more effective and can continue systemic therapy for longer periods (Table 3).

## 7. Cisplatin and Treatment of Cholangiocarcinoma

Cholangiocarcinoma (CCA) is an invasive malignant tumor arising from the biliary epithelium; intrahepatic CCA arising from the small intrahepatic bile duct upstream of the right and left hepatic ducts accounts for 20% [188]. Perihepatic CCA arising from second biliary ducts segmentation through common hepatic duct accounts for 50–60%, and distal CCA arising from common bile duct downstream from the confluence of the bile duct accounts for 20–30%, which were previously classified as extrahepatic CCA. CCA is a rare malignancy that accounts for approximately 3% of all digestive cancers, with an annual incidence of 2 per 100,000 people in Western countries, but has been reported to be on the rise in recent years [189]. Similar to HCC, intrahepatic CCC has also been associated with an increased incidence of chronic liver disease related to alcohol consumption and metabolic syndrome. CCA is largely unresectable (60–70%) at diagnosis, and systemic chemotherapy is indicated for systemic chemotherapy [188], although there are considerably fewer regimens recommended as first-line regimen. Owing to the complexity of early detection and few treatment options, the median overall survival of CCA was reported to be below 12 months and the 5-year survival rate below 5%, representing an extremely poor prognosis [190,191].

Randomized comparisons of gemcitabine (GEM) plus cisplatin (CIS) vs. GEM alone in the ABC-01 phase II and ABC-02 phase III trials showed a significant survival advantage with GEM/CIS therapy [192,193]. The ABC-02 phase III trial showed that GEM/CIS therapy was significantly superior to GEM alone, regardless of tumor stage or location, with a median overall survival (OS) of 11.7 and 8.1 months, respectively [193]. A comparative study in an Asian population using a similar regimen was conducted and showed favorable results with GEM/CIS therapy [194]. Since then, the first-line standard has been 24 weeks, or 8 cycles of CG therapy. In a phase II trial, a three-drug combination therapy (GCS therapy) with S-1 added to GEM/CIS therapy was tried, the results were excellent, with a response rate of 24% and a median OS of 16.2 months [195]. The KHBO1401 phase III trial comparing GCS and GEM/CIS therapy was subsequently conducted, proving the superiority of GCS therapy over GEM/CIS therapy [196]. GCS therapy is now being positioned as a new treatment option. In fact, several oncogenic drivers, i.e., FGFRs or isoforms 1 and 2 isocitrate dehydrogenase (IDH1/2), have recently been identified as potential useful therapeutic targets for CCA [197], but the usefulness of MTAs for CCA has not been well demonstrated. In combination therapy with cytotoxic agents and MTAs, the clinical study of GEM/CIS therapy plus cediranib, a vascular endothelial growth factor receptor inhibitor, was conducted but did not yield promising results [198]. Therefore, there have been limited anticancer drug regimens other than GEM/CIS therapy for first-line treatment of CCA, but with the advent of immunotherapy in 2022, that therapeutic strategy is about to undergo a breakthrough. In the phase III TOPAZ-1 trial, patients with advanced CCA were randomized to receive durvalumab, anti-PD-L1 inhibitor, or placebo for eight cycles combined with standard GEM/CIS therapy. The duravalumab group had significantly better OS, PFS, and objective response rate than the placebo group [199]. Interestingly, the combination of GEM/CIS therapy and durvalumab improved PFS when PD-L1 positivity in the tumor area was >1%, but had no significant impact on OS, indicating that it is important to identify biomarkers that predict response to immunotherapy. In addition, it is necessary to evaluate whether there is a long-term survival benefit typical of armored vehicles for cancer immunotherapy in the future.

The cellular mechanisms of GEM resistance to common cancers include altered drug metabolism, decreased drug accumulation in cancer cells, and activation of pro-survival pathways, which are common to CIS [163,200]. In addition, CIS resistance is enhanced by the activation of DNA damage repair [201]. By inhibiting Akt serine/threonine kinase activity, GEM can increase the retention of platinum drugs, such as CIS, owing to decreased DNA repair, making GEM/CIS therapy an excellent drug synergist [202]. In addition, CIS resistance can be reversed by combining GEM in several types of cancer [203,204]. Although GEM/CIS therapy is highly effective in the treatment of certain cases of CCA, drug resistance progresses rapidly in other cases. The resistance mechanism of GEM/CIS therapy is essential to enhancing its therapeutic effects. The recent study on GEM sensitivity to CCA showed that serum thrombospondin-1 (TSP1) could predict gemcitabine sensitivity in CCA patients, and in a functional analysis, TSP1 enhanced the effect of GEM [205]. Interestingly, a basic study that established GEM/CIS-resistant CCA cells and analyzed their resistance mechanism showed that resistant CCA acquired vulnerability to the molecular second mitochondrial activator of caspase (SMAC) mimetics, LCL161 and Birinapant, and was associated with increased expression of apoptosis inhibitory protein 2 (cIAP2), a known target of SMAC mimetics [206]. Analysis in xenograft models of GEM/CIS-resistant CCA cells also showed that LCL161 downregulated clAP2 expression and restored sensitivity to GEM/CIS, suggesting that the combination of LCL161 and GEM/CIS could prevent the emergence of drug resistance in CCA. Another study on N6-methyladenosine (m6 A) modification, which plays an important role in chemotherapy resistance, showed that CIS-resistant CCA tissue YTH domain family 2 (YTHDF2) expression was upregulated and correlated with poor prognosis [207]. YTHDF2 silencing caused cell cycle arrest and promoted apoptosis in cisplatin-resistant CCA cells, and decreased YHHDF2 expression restored cisplatin resistance in CCA cells. Although preclinical studies on chemotherapy for CCA are lacking, compared to those on other cancer types, elucidating the mechanisms of chemotherapy, particularly GEM/CIS, will not only provide biomarkers for selecting treatment regimens for patients with advanced CCA, but will contribute to therapeutic strategies, such as combination drugs, to overcome drug resistance.

## 8. Cisplatin for Pediatric Liver Tumors

Primary liver tumors account for 0.5–1.5% of tumors that occur in children [17] and are rare, with an incidence of approximately 1.6 cases per million children aged 0–14 years [208]. Hepatoblastoma (HB) is the most common (67–80%) type, followed by HCC, which accounts for 20–30% of the aforementioned cases [209]. HB generally develops in infancy, from 6 months to 3 years of age, and is the most common liver cancer among children under 3 years of age, decreasing after 5 years of age [209,210]. The male-to-female ratio is 1.6:1.0, with a predilection for boys [209,210]. The etiology of HB is unknown, but is associated with very low birth weight and various genetic disorders [210]. In contrast, most cases of HCC are adult-onset, with only a few pediatric cases, accounting for only 0.4 cases per million children aged 0–14 years [211]. Unlike adults, the etiology is unclear in most pediatric cases [208]. However, HCC occurring in underlying liver disease includes infections, such as HBV, and metabolic diseases, such as tyrosinemia and Alagille syndrome, with causes having regional variations depending on the prevalence of HBV [208,211,212].

Preoperative and postoperative chemotherapy and surgical approaches including hepatectomy and liver transplantation are effective in the treatment of HB and HCC [211]. HB combined with preoperative and postoperative chemotherapy has greatly improved outcomes [209]. In fact, the recurrence-free survival (EFS) and overall survival (OS) rates for HB are very high, ranging from about 30% in the 1970s to 70–90% in the 2010s [208]. Conversely, HCC is generally less sensitive to chemotherapy than HB [213], but preoperative chemotherapy may lower the stage of the cancer to a level where surgical resection is possible, and postoperative chemotherapy may minimize the risk of recurrence and metastasis [211]. Therefore, chemotherapy is a major determinant of the success of the surgical approach, and cisplatin is one of the key anticancer agents in that chemotherapy. Historically, HB and HCC have been treated with the same protocols. SIOPEL-1, the first other center trial by SIOPEL, included all HB patients introduced to PLADO (CDDP: 80 mg/m^2^, DXR 60 mg/m^2^) as preoperative chemotherapy, which showed a 5-year EFS of 66% and a favorable OS of 75% [214]. Moreover, 18 of 37 patients (49%) with HCC exhibited a partial response to PLADO, indicating that HCC can be sensitive to chemotherapy, unlike adult patients, but complete tumor resection was achieved in 14 of 39 patients (36%), and the 5-year EFS and OS accounted for 17% and 28% of the cases, respectively, which were not as good as the HB outcomes [215,216]. In subsequent SIOPEL-2 and SIOPEL-3 clinical trials, cisplatin-based regimens also showed remarkable OS and EFS in HB [217,218], but HCC did not show comparable efficacy to HB [217,218,219]. In addition, for such rare cancers, multicenter collaborative studies are essential to establishing high-quality evidence. Therefore, the Children’s Hepatic tumors International Collaboration (CHIC) was established in 2011, and is a collaboration of the Children Oncology Group (COG), Society of Liver Tumor Study Group (SIOPEL), the German Society of Pediatric Oncology and Hematology (GPOH), and the Japanese Study Group for Pediatric Liver Tumor (JPLT), the Pediatric Hepatitis International Treatment Trial (PHITT) was established to pursue optimal treatment [220,221].

The most problematic side effect of cisplatin in children is ototoxicity, the incidence of which ranges from 26% to more than 90% [222]. Cisplatin-induced hearing loss is generally bilateral, sensorineural, and permanent, and the degeneration of inner and outer hair cells of the cochlea involved in ototoxicity cannot be regenerated once damaged [223]. High frequencies (>4000 Hz) are initially affected, but may gradually progress to low frequencies (500–4000 Hz) necessary for understanding language, which may also affect language development [224]. Hearing loss is higher with higher cumulative doses of cisplatin or when treated from an early age [224]. Approximately 50% of children treated with cisplatin have some degree of permanent hearing loss, up to 90% have moderate-to-severe hearing loss, and up to 25% have severe hearing loss when cumulative doses exceed 400 mg/m^2^ [225]. Long-term surveillance is important in young patients until language development is complete, as ototoxicity can occur even after treatment is completed [226].

Although there is no clear prophylaxis for ototoxicity, the efficacy of sodium thiosulfate administration has been reported in several studies, with the addition of sodium thiosulfate reportedly reducing the risk of hearing loss by 48% [227,228]. Sodium thiosulfate acts as a reactive oxygen scavenger [224,228], and amifostine and D-methionine may be effective through a similar mechanism [224,229,230,231]. In clinical use, sodium thiosulfate is strongly recommended, particularly for non-metastatic hepatoblastoma [228]. Similar to the anticancer effects of cisplatin, the mechanism of ototoxicity is also thought to be due to cell cycle arrest and apoptosis caused by inhibition of DNA synthesis and RNA transcription associated with intra/inter-strand cross-linking of DNA strands, and cell death associated with caspase activation due to the production of ROS [229]. Therefore, local administration of auriculoprotective agents has generated great interest, dispelling concerns that they interfere with anticancer effects, and the efficacy of trans-ear drum administration of N-acetylcysteine is expected; however, lack of sufficient data has not led to a recommendation for clinical use [228].

## 9. Conclusions

Cisplatin, the first platinum-based compound approved as an anticancer drug, and other platinum-based compounds are the most effective in the treatment of various solid tumors, including hepatocellular carcinoma (HCC).

The most common mechanism by which platinum-based drugs exert their anticancer effects is by inducing cell death through inhibition of DNA replication and transcription. However, as with other anticancer drugs, they damage cancer and normal cells, and side effects to non-target organs and the acquisition of resistance remain critical problems, thus limiting their use. Various similar compounds have been developed to reduce side effects, and currently only two platinum drugs, carboplatin and oxaliplatin, other than cisplatin have been approved worldwide.

HCC is the most common primary liver tumor and is often diagnosed at an unresectable advanced stage. TACE and systemic chemotherapy are used to treat unresectable HCC. Systemic chemotherapy with doxorubicin or chemohormonal therapy with tamoxifen has also been investigated, but neither has demonstrated survival advantages. However, molecular-targeted agents and immune checkpoint inhibitors are effective as systemic therapy for unresectable HCC, and synergistic effects are expected when cisplatin is administered transhepatic arterially for treatment and then combined with systemic therapy.

## Figures and Tables

**Table 3:** Clinical trials of cisplatin in combination with molecular targeted agents and immune checkpoint inhibitors for HCC.

Molecular targeted agents		
Sorafenib	HCC with portal vein invasion	[178]
	Advanced HCC	[179]
	HCC with portal vein tumor thrombosis	[180]
	Advanced HCC	[181]
	Advanced HCC	[166]
	Unresectable HCC	[182]
	Advanced HCC	[183]
	Unresectable HCC	[184]
	Advanced HCC	[185]
	Pediatric HCC	[186]
Immune checkpoint inhibitors		
Atezolizumab	Advanced HCC	[187]

## Data Availability

Not applicable.

## References

[1] Omata M., Cheng A.L., Kokudo N., Kudo M., Lee J.M., Jia J., Tateishi R., Han K.H., Chawla Y.K., Shiina S. (2017). Asia-Pacific clinical practice guidelines on the management of hepatocellular carcinoma: A 2017 update. Hepatol. Int..

[2] European Association for the Study of the Liver (2018). EASL Clinical Practice Guidelines: Management of hepatocellular carcinoma. J. Hepatol..

[3] Marrero J.A., Kulik L.M., Sirlin C.B., Zhu A.X., Finn R.S., Abecassis M.M., Roberts L.R., Heimbach J.K. (2018). Diagnosis, Staging, and Management of Hepatocellular Carcinoma: 2018 Practice Guidance by the American Association for the Study of Liver Diseases. Hepatology.

[4] DeVita V.T., Chu E. (2008). A history of cancer chemotherapy. Cancer Res..

[5] Su S., Chen Y., Zhang P., Ma R., Zhang W., Liu J., Li T., Niu H., Cao Y., Hu B. (2022). The role of Platinum(IV)-based antitumor drugs and the anticancer immune response in medicinal inorganic chemistry. A systematic review from 2017 to 2022. Eur. J. Med. Chem..

[6] Ghosh S. (2019). Cisplatin: The first metal based anticancer drug. Bioorg. Chem..

[7] Szefler B., Czelen P. (2023). Will the Interactions of Some Platinum (II)-Based Drugs with B-Vitamins Reduce Their Therapeutic Effect in Cancer Patients? Comparison of Chemotherapeutic Agents such as Cisplatin, Carboplatin and Oxaliplatin-A Review. Int. J. Mol. Sci..

[8] Laface C., Laforgia M., Molinari P., Ugenti I., Gadaleta C.D., Porta C., Ranieri G. (2021). Hepatic Arterial Infusion of Chemotherapy for Advanced Hepatobiliary Cancers: State of the Art. Cancers.

[9] Laface C., Fedele P., Maselli F.M., Ambrogio F., Foti C., Molinari P., Ammendola M., Lioce M., Ranieri G. (2022). Targeted Therapy for Hepatocellular Carcinoma: Old and New Opportunities. Cancers.

[10] Laface C., Ranieri G., Maselli F.M., Ambrogio F., Foti C., Ammendola M., Laterza M., Cazzato G., Memeo R., Mastrandrea G. (2023). Immunotherapy and the Combination with Targeted Therapies for Advanced Hepatocellular Carcinoma. Cancers.

[11] Moawad A.W., Morshid A., Khalaf A.M., Elmohr M.M., Hazle J.D., Fuentes D., Badawy M., Kaseb A.O., Hassan M., Mahvash A. (2023). Multimodality annotated hepatocellular carcinoma data set including pre- and post-TACE with imaging segmentation. Sci. Data.

[12] Llovet J.M., Bruix J. (2003). Systematic review of randomized trials for unresectable hepatocellular carcinoma: Chemoembolization improves survival. Hepatology.

[13] Lopez P.M., Villanueva A., Llovet J.M. (2006). Systematic review: Evidence-based management of hepatocellular carcinoma—An updated analysis of randomized controlled trials. Aliment. Pharm. Ther..

[14] Llovet J.M., Ricci S., Mazzaferro V., Hilgard P., Gane E., Blanc J.F., de Oliveira A.C., Santoro A., Raoul J.L., Forner A. (2008). Sorafenib in advanced hepatocellular carcinoma. N. Engl. J. Med..

[15] Kudo M., Finn R.S., Qin S., Han K.H., Ikeda K., Piscaglia F., Baron A., Park J.W., Han G., Jassem J. (2018). Lenvatinib versus sorafenib in first-line treatment of patients with unresectable hepatocellular carcinoma: A randomised phase 3 non-inferiority trial. Lancet.

[16] Finn R.S., Qin S., Ikeda M., Galle P.R., Ducreux M., Kim T.Y., Kudo M., Breder V., Merle P., Kaseb A.O. (2020). Atezolizumab plus Bevacizumab in Unresectable Hepatocellular Carcinoma. N. Engl. J. Med..

[17] National Institute of Diabetes and Digestive and Kidney Diseases (2012). Platinum Coordination Complexes. LiverTox: Clinical and Research Information on Drug-Induced Liver Injury.

[18] Forgie B.N., Prakash R., Telleria C.M. (2022). Revisiting the Anti-Cancer Toxicity of Clinically Approved Platinating Derivatives. Int. J. Mol. Sci..

[19] Dasari S., Tchounwou P.B. (2014). Cisplatin in cancer therapy: Molecular mechanisms of action. Eur. J. Pharm..

[20] Rosenberg B., Vancamp L., Krigas T. (1965). Inhibition of Cell Division in Escherichia Coli by Electrolysis Products from a Platinum Electrode. Nature.

[21] Rosenberg B., VanCamp L., Trosko J.E., Mansour V.H. (1969). Platinum compounds: A new class of potent antitumour agents. Nature.

[22] Romani A.M.P. (2022). Cisplatin in cancer treatment. Biochem. Pharm..

[23] Kelland L. (2007). The resurgence of platinum-based cancer chemotherapy. Nat. Rev. Cancer.

[24] Ivanov A.I., Christodoulou J., Parkinson J.A., Barnham K.J., Tucker A., Woodrow J., Sadler P.J. (1998). Cisplatin binding sites on human albumin. J. Biol. Chem..

[25] Gullo J.J., Litterst C.L., Maguire P.J., Sikic B.I., Hoth D.F., Woolley P.V. (1980). Pharmacokinetics and protein binding of cis-dichlorodiammine platinum (II) administered as a one hour or as a twenty hour infusion. Cancer Chemother. Pharm..

[26] Makovec T. (2019). Cisplatin and beyond: Molecular mechanisms of action and drug resistance development in cancer chemotherapy. Radiol. Oncol..

[27] Eljack N.D., Ma H.Y., Drucker J., Shen C., Hambley T.W., New E.J., Friedrich T., Clarke R.J. (2014). Mechanisms of cell uptake and toxicity of the anticancer drug cisplatin. Metallomics.

[28] Lambert I.H., Sorensen B.H. (2018). Facilitating the Cellular Accumulation of Pt-Based Chemotherapeutic Drugs. Int. J. Mol. Sci..

[29] Kishimoto S., Yasuda M., Suzuki R., Fukushima S. (2016). Intracellular uptake of an antitumor-active azole-bridged dinuclear platinum(II) complex in cisplatin-resistant tumor cells. Biometals.

[30] Binks S.P., Dobrota M. (1990). Kinetics and mechanism of uptake of platinum-based pharmaceuticals by the rat small intestine. Biochem. Pharm..

[31] Yonezawa A., Masuda S., Yokoo S., Katsura T., Inui K. (2006). Cisplatin and oxaliplatin, but not carboplatin and nedaplatin, are substrates for human organic cation transporters (SLC22A1-3 and multidrug and toxin extrusion family). J. Pharm. Exp. Ther..

[32] Nieskens T.T.G., Peters J.G.P., Dabaghie D., Korte D., Jansen K., Van Asbeck A.H., Tavraz N.N., Friedrich T., Russel F.G.M., Masereeuw R. (2018). Expression of Organic Anion Transporter 1 or 3 in Human Kidney Proximal Tubule Cells Reduces Cisplatin Sensitivity. Drug Metab. Dispos..

[33] Wee N.K., Weinstein D.C., Fraser S.T., Assinder S.J. (2013). The mammalian copper transporters CTR1 and CTR2 and their roles in development and disease. Int. J. Biochem. Cell Biol..

[34] Ishida S., Lee J., Thiele D.J., Herskowitz I. (2002). Uptake of the anticancer drug cisplatin mediated by the copper transporter Ctr1 in yeast and mammals. Proc. Natl. Acad. Sci. USA.

[35] Safaei R. (2006). Role of copper transporters in the uptake and efflux of platinum containing drugs. Cancer Lett..

[36] Kuo M.T., Fu S., Savaraj N., Chen H.H. (2012). Role of the human high-affinity copper transporter in copper homeostasis regulation and cisplatin sensitivity in cancer chemotherapy. Cancer Res..

[37] Katano K., Kondo A., Safaei R., Holzer A., Samimi G., Mishima M., Kuo Y.M., Rochdi M., Howell S.B. (2002). Acquisition of resistance to cisplatin is accompanied by changes in the cellular pharmacology of copper. Cancer Res..

[38] Ivy K.D., Kaplan J.H. (2013). A re-evaluation of the role of hCTR1, the human high-affinity copper transporter, in platinum-drug entry into human cells. Mol. Pharm..

[39] Beretta G.L., Gatti L., Tinelli S., Corna E., Colangelo D., Zunino F., Perego P. (2004). Cellular pharmacology of cisplatin in relation to the expression of human copper transporter CTR1 in different pairs of cisplatin-sensitive and-resistant cells. Biochem. Pharm..

[40] Basu A., Krishnamurthy S. (2010). Cellular responses to Cisplatin-induced DNA damage. J. Nucleic Acids.

[41] Lugones Y., Loren P., Salazar L.A. (2022). Cisplatin Resistance: Genetic and Epigenetic Factors Involved. Biomolecules.

[42] Plooy A.C., Fichtinger-Schepman A.M., Schutte H.H., van Dijk M., Lohman P.H. (1985). The quantitative detection of various Pt-DNA-adducts in Chinese hamster ovary cells treated with cisplatin: Application of immunochemical techniques. Carcinogenesis.

[43] Eastman A. (1986). Reevaluation of interaction of cis-dichloro(ethylenediamine)platinum(II) with DNA. Biochemistry.

[44] Deans A.J., West S.C. (2011). DNA interstrand crosslink repair and cancer. Nat. Rev. Cancer.

[45] Florea A.M., Busselberg D. (2011). Cisplatin as an anti-tumor drug: Cellular mechanisms of activity, drug resistance and induced side effects. Cancers.

[46] Ranasinghe R., Mathai M.L., Zulli A. (2022). Cisplatin for cancer therapy and overcoming chemoresistance. Heliyon.

[47] Vichi P., Coin F., Renaud J.P., Vermeulen W., Hoeijmakers J.H., Moras D., Egly J.M. (1997). Cisplatin- and UV-damaged DNA lure the basal transcription factor TFIID/TBP. EMBO J..

[48] Treiber D.K., Zhai X., Jantzen H.M., Essigmann J.M. (1994). Cisplatin-DNA adducts are molecular decoys for the ribosomal RNA transcription factor hUBF (human upstream binding factor). Proc. Natl. Acad. Sci. USA.

[49] Zhai X., Beckmann H., Jantzen H.M., Essigmann J.M. (1998). Cisplatin-DNA adducts inhibit ribosomal RNA synthesis by hijacking the transcription factor human upstream binding factor. Biochemistry.

[50] Mymryk J.S., Zaniewski E., Archer T.K. (1995). Cisplatin inhibits chromatin remodeling, transcription factor binding, and transcription from the mouse mammary tumor virus promoter in vivo. Proc. Natl. Acad. Sci. USA.

[51] Ober M., Lippard S.J. (2008). A 1,2-d(GpG) cisplatin intrastrand cross-link influences the rotational and translational setting of DNA in nucleosomes. J. Am. Chem. Soc..

[52] Todd R.C., Lippard S.J. (2010). Consequences of cisplatin binding on nucleosome structure and dynamics. Chem. Biol..

[53] Zhu G., Song L., Lippard S.J. (2013). Visualizing inhibition of nucleosome mobility and transcription by cisplatin-DNA interstrand crosslinks in live mammalian cells. Cancer Res..

[54] Achkar I.W., Abdulrahman N., Al-Sulaiti H., Joseph J.M., Uddin S., Mraiche F. (2018). Cisplatin based therapy: The role of the mitogen activated protein kinase signaling pathway. J. Transl. Med..

[55] Olivero O.A., Chang P.K., Lopez-Larraza D.M., Semino-Mora M.C., Poirier M.C. (1997). Preferential formation and decreased removal of cisplatin-DNA adducts in Chinese hamster ovary cell mitochondrial DNA as compared to nuclear DNA. Mutat. Res..

[56] Yang Z., Schumaker L.M., Egorin M.J., Zuhowski E.G., Guo Z., Cullen K.J. (2006). Cisplatin preferentially binds mitochondrial DNA and voltage-dependent anion channel protein in the mitochondrial membrane of head and neck squamous cell carcinoma: Possible role in apoptosis. Clin. Cancer Res..

[57] Redza-Dutordoir M., Averill-Bates D.A. (2016). Activation of apoptosis signalling pathways by reactive oxygen species. Biochim. Biophys. Acta.

[58] Marullo R., Werner E., Degtyareva N., Moore B., Altavilla G., Ramalingam S.S., Doetsch P.W. (2013). Cisplatin induces a mitochondrial-ROS response that contributes to cytotoxicity depending on mitochondrial redox status and bioenergetic functions. PLoS ONE.

[59] Gupta S.C., Hevia D., Patchva S., Park B., Koh W., Aggarwal B.B. (2012). Upsides and downsides of reactive oxygen species for cancer: The roles of reactive oxygen species in tumorigenesis, prevention, and therapy. Antioxid. Redox Signal..

[60] Hampton M.B., Orrenius S. (1997). Dual regulation of caspase activity by hydrogen peroxide: Implications for apoptosis. FEBS Lett..

[61] Shrivastava A., Kuzontkoski P.M., Groopman J.E., Prasad A. (2011). Cannabidiol induces programmed cell death in breast cancer cells by coordinating the cross-talk between apoptosis and autophagy. Mol. Cancer Ther..

[62] Shen D.W., Pouliot L.M., Hall M.D., Gottesman M.M. (2012). Cisplatin resistance: A cellular self-defense mechanism resulting from multiple epigenetic and genetic changes. Pharm. Rev..

[63] Kalayda G.V., Wagner C.H., Jaehde U. (2012). Relevance of copper transporter 1 for cisplatin resistance in human ovarian carcinoma cells. J. Inorg. Biochem..

[64] Kilari D., Guancial E., Kim E.S. (2016). Role of copper transporters in platinum resistance. World J. Clin. Oncol..

[65] Korita P.V., Wakai T., Shirai Y., Matsuda Y., Sakata J., Takamura M., Yano M., Sanpei A., Aoyagi Y., Hatakeyama K. (2010). Multidrug resistance-associated protein 2 determines the efficacy of cisplatin in patients with hepatocellular carcinoma. Oncol. Rep..

[66] Pearson S.A., Cowan J.A. (2021). Glutathione-coordinated metal complexes as substrates for cellular transporters. Metallomics.

[67] Chen H.H., Kuo M.T. (2010). Role of glutathione in the regulation of Cisplatin resistance in cancer chemotherapy. Met. Based Drugs.

[68] Hinoshita E., Uchiumi T., Taguchi K., Kinukawa N., Tsuneyoshi M., Maehara Y., Sugimachi K., Kuwano M. (2000). Increased expression of an ATP-binding cassette superfamily transporter, multidrug resistance protein 2, in human colorectal carcinomas. Clin. Cancer Res..

[69] Byun S.S., Kim S.W., Choi H., Lee C., Lee E. (2005). Augmentation of cisplatin sensitivity in cisplatin-resistant human bladder cancer cells by modulating glutathione concentrations and glutathione-related enzyme activities. BJU Int..

[70] Rocha C.R., Garcia C.C., Vieira D.B., Quinet A., de Andrade-Lima L.C., Munford V., Belizario J.E., Menck C.F. (2014). Glutathione depletion sensitizes cisplatin- and temozolomide-resistant glioma cells in vitro and in vivo. Cell Death Dis..

[71] Konoshenko M., Lansukhay Y., Krasilnikov S., Laktionov P. (2022). MicroRNAs as Predictors of Lung-Cancer Resistance and Sensitivity to Cisplatin. Int. J. Mol. Sci..

[72] Zou Y., Liu Y., Wu X., Shell S.M. (2006). Functions of human replication protein A (RPA): From DNA replication to DNA damage and stress responses. J. Cell Physiol..

[73] Nasrallah N.A., Wiese B.M., Sears C.R. (2022). Xeroderma Pigmentosum Complementation Group C (XPC): Emerging Roles in Non-Dermatologic Malignancies. Front Oncol.

[74] Fang C., Chen Y.X., Wu N.Y., Yin J.Y., Li X.P., Huang H.S., Zhang W., Zhou H.H., Liu Z.Q. (2017). MiR-488 inhibits proliferation and cisplatin sensibility in non-small-cell lung cancer (NSCLC) cells by activating the eIF3a-mediated NER signaling pathway. Sci. Rep..

[75] Farmer H., McCabe N., Lord C.J., Tutt A.N., Johnson D.A., Richardson T.B., Santarosa M., Dillon K.J., Hickson I., Knights C. (2005). Targeting the DNA repair defect in BRCA mutant cells as a therapeutic strategy. Nature.

[76] Sakai W., Swisher E.M., Karlan B.Y., Agarwal M.K., Higgins J., Friedman C., Villegas E., Jacquemont C., Farrugia D.J., Couch F.J. (2008). Secondary mutations as a mechanism of cisplatin resistance in BRCA2-mutated cancers. Nature.

[77] Swisher E.M., Sakai W., Karlan B.Y., Wurz K., Urban N., Taniguchi T. (2008). Secondary BRCA1 mutations in BRCA1-mutated ovarian carcinomas with platinum resistance. Cancer Res..

[78] Zhao J., Fu W., Liao H., Dai L., Jiang Z., Pan Y., Huang H., Mo Y., Li S., Yang G. (2015). The regulatory and predictive functions of miR-17 and miR-92 families on cisplatin resistance of non-small cell lung cancer. BMC Cancer.

[79] Nehme A., Baskaran R., Nebel S., Fink D., Howell S.B., Wang J.Y., Christen R.D. (1999). Induction of JNK and c-Abl signalling by cisplatin and oxaliplatin in mismatch repair-proficient and -deficient cells. Br. J. Cancer.

[80] Martinez-Rivera M., Siddik Z.H. (2012). Resistance and gain-of-resistance phenotypes in cancers harboring wild-type p53. Biochem. Pharm..

[81] Tsang R.Y., Al-Fayea T., Au H.J. (2009). Cisplatin overdose: Toxicities and management. Drug Saf..

[82] Ali R., Aouida M., Alhaj Sulaiman A., Madhusudan S., Ramotar D. (2022). Can Cisplatin Therapy Be Improved? Pathways That Can Be Targeted. Int. J. Mol. Sci..

[83] Crona D.J., Faso A., Nishijima T.F., McGraw K.A., Galsky M.D., Milowsky M.I. (2017). A Systematic Review of Strategies to Prevent Cisplatin-Induced Nephrotoxicity. Oncologist.

[84] Volarevic V., Djokovic B., Jankovic M.G., Harrell C.R., Fellabaum C., Djonov V., Arsenijevic N. (2019). Molecular mechanisms of cisplatin-induced nephrotoxicity: A balance on the knife edge between renoprotection and tumor toxicity. J. Biomed. Sci..

[85] Fang C.Y., Lou D.Y., Zhou L.Q., Wang J.C., Yang B., He Q.J., Wang J.J., Weng Q.J. (2021). Natural products: Potential treatments for cisplatin-induced nephrotoxicity. Acta Pharm. Sin..

[86] Yao X., Panichpisal K., Kurtzman N., Nugent K. (2007). Cisplatin nephrotoxicity: A review. Am. J. Med. Sci..

[87] Pabla N., Murphy R.F., Liu K., Dong Z. (2009). The copper transporter Ctr1 contributes to cisplatin uptake by renal tubular cells during cisplatin nephrotoxicity. Am. J. Physiol. Ren. Physiol..

[88] Filipski K.K., Loos W.J., Verweij J., Sparreboom A. (2008). Interaction of Cisplatin with the human organic cation transporter 2. Clin. Cancer Res..

[89] Filipski K.K., Mathijssen R.H., Mikkelsen T.S., Schinkel A.H., Sparreboom A. (2009). Contribution of organic cation transporter 2 (OCT2) to cisplatin-induced nephrotoxicity. Clin. Pharm. Ther..

[90] Miller R.P., Tadagavadi R.K., Ramesh G., Reeves W.B. (2010). Mechanisms of Cisplatin nephrotoxicity. Toxins.

[91] Deng F., Sharma I., Dai Y., Yang M., Kanwar Y.S. (2019). Myo-inositol oxygenase expression profile modulates pathogenic ferroptosis in the renal proximal tubule. J. Clin. Investig..

[92] Baliga R., Ueda N., Walker P.D., Shah S.V. (1999). Oxidant mechanisms in toxic acute renal failure. Drug Metab. Rev..

[93] Yang C., Kaushal V., Haun R.S., Seth R., Shah S.V., Kaushal G.P. (2008). Transcriptional activation of caspase-6 and -7 genes by cisplatin-induced p53 and its functional significance in cisplatin nephrotoxicity. Cell Death Differ..

[94] Tsuruya K., Ninomiya T., Tokumoto M., Hirakawa M., Masutani K., Taniguchi M., Fukuda K., Kanai H., Kishihara K., Hirakata H. (2003). Direct involvement of the receptor-mediated apoptotic pathways in cisplatin-induced renal tubular cell death. Kidney Int..

[95] Ramesh G., Reeves W.B. (2002). TNF-alpha mediates chemokine and cytokine expression and renal injury in cisplatin nephrotoxicity. J. Clin. Investig..

[96] Dong Z., Atherton S.S. (2007). Tumor necrosis factor-alpha in cisplatin nephrotoxicity: A homebred foe?. Kidney Int..

[97] Zhang B., Ramesh G., Norbury C.C., Reeves W.B. (2007). Cisplatin-induced nephrotoxicity is mediated by tumor necrosis factor-alpha produced by renal parenchymal cells. Kidney Int..

[98] Liu M., Chien C.C., Burne-Taney M., Molls R.R., Racusen L.C., Colvin R.B., Rabb H. (2006). A pathophysiologic role for T lymphocytes in murine acute cisplatin nephrotoxicity. J. Am. Soc. Nephrol..

[99] Tiseo M., Martelli O., Mancuso A., Sormani M.P., Bruzzi P., Di Salvia R., De Marinis F., Ardizzoni A. (2007). Short hydration regimen and nephrotoxicity of intermediate to high-dose cisplatin-based chemotherapy for outpatient treatment in lung cancer and mesothelioma. Tumori.

[100] Lavole A., Danel S., Baudrin L., Gounant V., Ruppert A.M., Epaud C., Belmont L., Rosencher L., Cadranel J., Milleron B. (2012). Routine administration of a single dose of cisplatin >/= 75 mg/m^2^ after short hydration in an outpatient lung-cancer clinic. Bull. Cancer.

[101] Ouchi A., Asano M., Aono K., Watanabe T., Kato T. (2014). Comparison of short and continuous hydration regimen in chemotherapy containing intermediate- to high-dose Cisplatin. J. Oncol..

[102] Ninomiya K., Hotta K., Hisamoto-Sato A., Ichihara E., Gotoda H., Morichika D., Tamura T., Kayatani H., Minami D., Kubo T. (2016). Short-term low-volume hydration in cisplatin-based chemotherapy for patients with lung cancer: The second prospective feasibility study in the Okayama Lung Cancer Study Group Trial 1201. Int. J. Clin. Oncol..

[103] Hase T., Miyazaki M., Ichikawa K., Yogo N., Ozawa N., Hatta T., Ando M., Sato M., Kondo M., Yamada K. (2020). Short hydration with 20 mEq of magnesium supplementation for lung cancer patients receiving cisplatin-based chemotherapy: A prospective study. Int. J. Clin. Oncol..

[104] Yamamoto Y., Watanabe K., Tsukiyama I., Yabushita H., Matsuura K., Wakatsuki A. (2016). Hydration with 15 mEq Magnesium Is Effective at Reducingthe Risk for Cisplatin-induced Nephrotoxicity in Patients Receiving Cisplatin (>/=50 mg/m^2^) Combination Chemotherapy. Anticancer Res..

[105] Miyoshi T., Hayashi T., Uoi M., Omura F., Tsumagari K., Maesaki S., Yokota C., Nakano T., Egawa T. (2022). Preventive effect of 20 mEq and 8 mEq magnesium supplementation on cisplatin-induced nephrotoxicity: A propensity score-matched analysis. Support Care Cancer.

[106] Casanova A.G., Hernandez-Sanchez M.T., Lopez-Hernandez F.J., Martinez-Salgado C., Prieto M., Vicente-Vicente L., Morales A.I. (2020). Systematic review and meta-analysis of the efficacy of clinically tested protectants of cisplatin nephrotoxicity. Eur. J. Clin. Pharm..

[107] Yokoo K., Murakami R., Matsuzaki T., Yoshitome K., Hamada A., Saito H. (2009). Enhanced renal accumulation of cisplatin via renal organic cation transporter deteriorates acute kidney injury in hypomagnesemic rats. Clin. Exp. Nephrol..

[108] Solanki M.H., Chatterjee P.K., Xue X., Gupta M., Rosales I., Yeboah M.M., Kohn N., Metz C.N. (2015). Magnesium protects against cisplatin-induced acute kidney injury without compromising cisplatin-mediated killing of an ovarian tumor xenograft in mice. Am. J. Physiol. Renal Physiol..

[109] Solanki M.H., Chatterjee P.K., Gupta M., Xue X., Plagov A., Metz M.H., Mintz R., Singhal P.C., Metz C.N. (2014). Magnesium protects against cisplatin-induced acute kidney injury by regulating platinum accumulation. Am. J. Physiol. Renal Physiol..

[110] Ruggiero A., Rizzo D., Trombatore G., Maurizi P., Riccardi R. (2016). The ability of mannitol to decrease cisplatin-induced nephrotoxicity in children: Real or not?. Cancer Chemother. Pharm..

[111] Sainamthip P., Saichaemchan S., Satirapoj B., Prasongsook N. (2022). The Effect of Intravenous Mannitol Combined With Normal Saline in Preventing Cisplatin-Induced Nephrotoxicity: A Randomized, Double-Blind, Placebo-Controlled Trial. JCO Glob. Oncol..

[112] McKibbin T., Cheng L.L., Kim S., Steuer C.E., Owonikoko T.K., Khuri F.R., Shin D.M., Saba N.F. (2016). Mannitol to prevent cisplatin-induced nephrotoxicity in patients with squamous cell cancer of the head and neck (SCCHN) receiving concurrent therapy. Support Care Cancer.

[113] Morgan K.P., Buie L.W., Savage S.W. (2012). The role of mannitol as a nephroprotectant in patients receiving cisplatin therapy. Ann. Pharmacother..

[114] Ruggiero A., Ariano A., Triarico S., Capozza M.A., Romano A., Maurizi P., Mastrangelo S., Attina G. (2021). Cisplatin-induced nephrotoxicity in children: What is the best protective strategy?. J. Oncol. Pharm. Pract..

[115] Callejo A., Sedo-Cabezon L., Juan I.D., Llorens J. (2015). Cisplatin-Induced Ototoxicity: Effects, Mechanisms and Protection Strategies. Toxics.

[116] Rybak L.P., Mukherjea D., Ramkumar V. (2019). Mechanisms of Cisplatin-Induced Ototoxicity and Prevention. Semin. Hear..

[117] Zajaczkowska R., Kocot-Kepska M., Leppert W., Wrzosek A., Mika J., Wordliczek J. (2019). Mechanisms of Chemotherapy-Induced Peripheral Neuropathy. Int. J. Mol. Sci..

[118] Brouwers E.E., Huitema A.D., Boogerd W., Beijnen J.H., Schellens J.H. (2009). Persistent neuropathy after treatment with cisplatin and oxaliplatin. Acta Oncol..

[119] Gregg R.W., Molepo J.M., Monpetit V.J., Mikael N.Z., Redmond D., Gadia M., Stewart D.J. (1992). Cisplatin neurotoxicity: The relationship between dosage, time, and platinum concentration in neurologic tissues, and morphologic evidence of toxicity. J. Clin. Oncol..

[120] Starobova H., Vetter I. (2017). Pathophysiology of Chemotherapy-Induced Peripheral Neuropathy. Front. Mol. Neurosci..

[121] Liu M., Liu J., Wang L., Wu H., Zhou C., Zhu H., Xu N., Xie Y. (2014). Association of serum microRNA expression in hepatocellular carcinomas treated with transarterial chemoembolization and patient survival. PLoS ONE.

[122] Kerckhove N., Collin A., Conde S., Chaleteix C., Pezet D., Balayssac D. (2017). Long-Term Effects, Pathophysiological Mechanisms, and Risk Factors of Chemotherapy-Induced Peripheral Neuropathies: A Comprehensive Literature Review. Front. Pharm..

[123] Santos N., Ferreira R.S., Santos A.C.D. (2020). Overview of cisplatin-induced neurotoxicity and ototoxicity, and the protective agents. Food Chem. Toxicol..

[124] Olthoff K.M., Rosove M.H., Shackleton C.R., Imagawa D.K., Farmer D.G., Northcross P., Pakrasi A.L., Martin P., Goldstein L.I., Shaked A. (1995). Adjuvant chemotherapy improves survival after liver transplantation for hepatocellular carcinoma. Ann. Surg..

[125] Astolfi L., Ghiselli S., Guaran V., Chicca M., Simoni E., Olivetto E., Lelli G., Martini A. (2013). Correlation of adverse effects of cisplatin administration in patients affected by solid tumours: A retrospective evaluation. Oncol. Rep..

[126] Al-Malki A.L., Sayed A.A. (2014). Thymoquinone attenuates cisplatin-induced hepatotoxicity via nuclear factor kappa-beta. BMC Complement Altern Med..

[127] Hu Y., Sun B., Zhao B., Mei D., Gu Q., Tian Z. (2018). Cisplatin-induced cardiotoxicity with midrange ejection fraction: A case report and review of the literature. Medicine.

[128] Sung H., Ferlay J., Siegel R.L., Laversanne M., Soerjomataram I., Jemal A., Bray F. (2021). Global Cancer Statistics 2020: GLOBOCAN Estimates of Incidence and Mortality Worldwide for 36 Cancers in 185 Countries. CA Cancer J. Clin..

[129] Vogel A., Meyer T., Sapisochin G., Salem R., Saborowski A. (2022). Hepatocellular carcinoma. Lancet.

[130] Petrick J.L., Florio A.A., Znaor A., Ruggieri D., Laversanne M., Alvarez C.S., Ferlay J., Valery P.C., Bray F., McGlynn K.A. (2020). International trends in hepatocellular carcinoma incidence, 1978-2012. Int. J. Cancer.

[131] Younossi Z.M. (2019). Non-alcoholic fatty liver disease—A global public health perspective. J. Hepatol..

[132] Manjunatha N., Ganduri V., Rajasekaran K., Duraiyarasan S., Adefuye M. (2022). Transarterial Chemoembolization and Unresectable Hepatocellular Carcinoma: A Narrative Review. Cureus.

[133] Kasai K., Ushio A., Kasai Y., Sawara K., Miyamoto Y., Oikawa K., Takikawa Y., Suzuki K. (2013). Therapeutic efficacy of transarterial chemo-embolization with a fine-powder formulation of cisplatin for hepatocellular carcinoma. World J. Gastroenterol..

[134] Yodono H., Matsuo K., Shinohara A. (2011). A retrospective comparative study of epirubicin-lipiodol emulsion and cisplatin-lipiodol suspension for use with transcatheter arterial chemoembolization for treatment of hepatocellular carcinoma. Anticancer Drugs.

[135] Maeda N., Osuga K., Higashihara H., Tomoda K., Mikami K., Nakazawa T., Nakamura H., Tomiyama N. (2012). Transarterial chemoembolization with cisplatin as second-line treatment for hepatocellular carcinoma unresponsive to chemoembolization with epirubicin-Lipiodol emulsion. Cardiovasc. Interv. Radiol..

[136] Kamada K., Nakanishi T., Kitamoto M., Aikata H., Kawakami Y., Ito K., Asahara T., Kajiyama G. (2001). Long-term prognosis of patients undergoing transcatheter arterial chemoembolization for unresectable hepatocellular carcinoma: Comparison of cisplatin lipiodol suspension and doxorubicin hydrochloride emulsion. J. Vasc. Interv. Radiol..

[137] De Baere T., Ronot M., Chung J.W., Golfieri R., Kloeckner R., Park J.W., Gebauer B., Kibriya N., Ananthakrishnan G., Miyayama S. (2022). Initiative on Superselective Conventional Transarterial Chemoembolization Results (INSPIRE). Cardiovasc. Interv. Radiol..

[138] Galle P.R., Tovoli F., Foerster F., Worns M.A., Cucchetti A., Bolondi L. (2017). The treatment of intermediate stage tumours beyond TACE: From surgery to systemic therapy. J. Hepatol..

[139] Kudo M., Ueshima K., Chan S., Minami T., Chishina H., Aoki T., Takita M., Hagiwara S., Minami Y., Ida H. (2019). Lenvatinib as an Initial Treatment in Patients with Intermediate-Stage Hepatocellular Carcinoma Beyond Up-To-Seven Criteria and Child-Pugh A Liver Function: A Proof-Of-Concept Study. Cancers.

[140] Reig M., Forner A., Rimola J., Ferrer-Fabrega J., Burrel M., Garcia-Criado A., Kelley R.K., Galle P.R., Mazzaferro V., Salem R. (2022). BCLC strategy for prognosis prediction and treatment recommendation: The 2022 update. J. Hepatol..

[141] Park J.Y., Ahn S.H., Yoon Y.J., Kim J.K., Lee H.W., Lee D.Y., Chon C.Y., Moon Y.M., Han K.H. (2007). Repetitive short-course hepatic arterial infusion chemotherapy with high-dose 5-fluorouracil and cisplatin in patients with advanced hepatocellular carcinoma. Cancer.

[142] Kim B.K., Park J.Y., Choi H.J., Kim D.Y., Ahn S.H., Kim J.K., Lee D.Y., Lee K.H., Han K.H. (2011). Long-term clinical outcomes of hepatic arterial infusion chemotherapy with cisplatin with or without 5-fluorouracil in locally advanced hepatocellular carcinoma. J. Cancer Res. Clin. Oncol..

[143] Long G.B., Xiao C.W., Zhao X.Y., Zhang J., Li X. (2020). Effects of hepatic arterial infusion chemotherapy in the treatment of hepatocellular carcinoma: A meta-analysis. Medicine.

[144] Obi S., Sato S., Kawai T. (2015). Current Status of Hepatic Arterial Infusion Chemotherapy. Liver Cancer.

[145] Shim J.H., Park J.W., Kim J.H., An M., Kong S.Y., Nam B.H., Choi J.I., Kim H.B., Lee W.J., Kim C.M. (2008). Association between increment of serum VEGF level and prognosis after transcatheter arterial chemoembolization in hepatocellular carcinoma patients. Cancer Sci..

[146] Pezzuto A., Carico E. (2018). Role of HIF-1 in Cancer Progression: Novel Insights. A Review. Curr. Mol. Med..

[147] Huang Y., Lin D., Taniguchi C.M. (2017). Hypoxia inducible factor (HIF) in the tumor microenvironment: Friend or foe?. Sci. China Life Sci..

[148] Xiong Z.P., Yang S.R., Liang Z.Y., Xiao E.H., Yu X.P., Zhou S.K., Zhang Z.S. (2004). Association between vascular endothelial growth factor and metastasis after transcatheter arterial chemoembolization in patients with hepatocellular carcinoma. Hepatobiliary Pancreat Dis. Int..

[149] Hsieh M.Y., Lin Z.Y., Chuang W.L. (2011). Serial serum VEGF-A, angiopoietin-2, and endostatin measurements in cirrhotic patients with hepatocellular carcinoma treated by transcatheter arterial chemoembolization. Kaohsiung J. Med. Sci..

[150] Liu K., Min X.L., Peng J., Yang K., Yang L., Zhang X.M. (2016). The Changes of HIF-1alpha and VEGF Expression After TACE in Patients With Hepatocellular Carcinoma. J. Clin. Med. Res..

[151] Wei X., Zhao L., Ren R., Ji F., Xue S., Zhang J., Liu Z., Ma Z., Wang X.W., Wong L. (2021). MiR-125b Loss Activated HIF1alpha/pAKT Loop, Leading to Transarterial Chemoembolization Resistance in Hepatocellular Carcinoma. Hepatology.

[152] Ali H.E.A., Emam A.A., Zeeneldin A.A., Srour R., Tabashy R., El-Desouky E.D., Abd Elmageed Z.Y., Abdel-Wahab A.A. (2019). Circulating miR-26a, miR-106b, miR-107 and miR-133b stratify hepatocellular carcinoma patients according to their response to transarterial chemoembolization. Clin. Biochem..

[153] Suehiro T., Miyaaki H., Kanda Y., Shibata H., Honda T., Ozawa E., Miuma S., Taura N., Nakao K. (2018). Serum exosomal microRNA-122 and microRNA-21 as predictive biomarkers in transarterial chemoembolization-treated hepatocellular carcinoma patients. Oncol. Lett..

[154] Qin L., Zhan Z., Wei C., Li X., Zhang T., Li J. (2021). Hsa-circRNA-G004213 promotes cisplatin sensitivity by regulating miR-513b-5p/PRPF39 in liver cancer. Mol. Med. Rep..

[155] Shi X., Wang B., Feng X., Xu Y., Lu K., Sun M. (2020). circRNAs and Exosomes: A Mysterious Frontier for Human Cancer. Mol. Ther. Nucleic. Acids.

[156] Cheng A.L., Kang Y.K., Chen Z., Tsao C.J., Qin S., Kim J.S., Luo R., Feng J., Ye S., Yang T.S. (2009). Efficacy and safety of sorafenib in patients in the Asia-Pacific region with advanced hepatocellular carcinoma: A phase III randomised, double-blind, placebo-controlled trial. Lancet Oncol..

[157] Bruix J., Qin S., Merle P., Granito A., Huang Y.H., Bodoky G., Pracht M., Yokosuka O., Rosmorduc O., Breder V. (2017). Regorafenib for patients with hepatocellular carcinoma who progressed on sorafenib treatment (RESORCE): A randomised, double-blind, placebo-controlled, phase 3 trial. Lancet.

[158] Abou-Alfa G.K., Meyer T., Cheng A.L., El-Khoueiry A.B., Rimassa L., Ryoo B.Y., Cicin I., Merle P., Chen Y., Park J.W. (2018). Cabozantinib in Patients with Advanced and Progressing Hepatocellular Carcinoma. N. Engl. J. Med..

[159] Zhu A.X., Kang Y.K., Yen C.J., Finn R.S., Galle P.R., Llovet J.M., Assenat E., Brandi G., Pracht M., Lim H.Y. (2019). Ramucirumab after sorafenib in patients with advanced hepatocellular carcinoma and increased alpha-fetoprotein concentrations (REACH-2): A randomised, double-blind, placebo-controlled, phase 3 trial. Lancet Oncol..

[160] Kudo M., Ueshima K., Ikeda M., Torimura T., Tanabe N., Aikata H., Izumi N., Yamasaki T., Nojiri S., Hino K. (2022). Final Results of TACTICS: A Randomized, Prospective Trial Comparing Transarterial Chemoembolization Plus Sorafenib to Transarterial Chemoembolization Alone in Patients with Unresectable Hepatocellular Carcinoma. Liver Cancer.

[161] Kuroda H., Oikawa T., Ninomiya M., Fujita M., Abe K., Okumoto K., Katsumi T., Sato W., Igarashi G., Iino C. (2022). Objective Response by mRECIST to Initial Lenvatinib Therapy Is an Independent Factor Contributing to Deep Response in Hepatocellular Carcinoma Treated with Lenvatinib-Transcatheter Arterial Chemoembolization Sequential Therapy. Liver Cancer.

[162] Kudo M. (2019). A New Treatment Option for Intermediate-Stage Hepatocellular Carcinoma with High Tumor Burden: Initial Lenvatinib Therapy with Subsequent Selective TACE. Liver Cancer.

[163] Jain A., Bhardwaj V. (2021). Therapeutic resistance in pancreatic ductal adenocarcinoma: Current challenges and future opportunities. World J. Gastroenterol..

[164] Sun Y., Zhang W., Bi X., Yang Z., Tang Y., Jiang L., Bi F., Chen M., Cheng S., Chi Y. (2022). Systemic Therapy for Hepatocellular Carcinoma: Chinese Consensus-Based Interdisciplinary Expert Statements. Liver Cancer.

[165] Granito A., Marinelli S., Forgione A., Renzulli M., Benevento F., Piscaglia F., Tovoli F. (2021). Regorafenib Combined with Other Systemic Therapies: Exploring Promising Therapeutic Combinations in HCC. J. Hepatocell. Carcinoma.

[166] Kudo M., Ueshima K., Yokosuka O., Ogasawara S., Obi S., Izumi N., Aikata H., Nagano H., Hatano E., Sasaki Y. (2018). Sorafenib plus low-dose cisplatin and fluorouracil hepatic arterial infusion chemotherapy versus sorafenib alone in patients with advanced hepatocellular carcinoma (SILIUS): A randomised, open label, phase 3 trial. Lancet Gastroenterol. Hepatol..

[167] Hamaya S., Fujihara S., Iwama H., Fujita K., Shi T., Nakabayashi R., Mizuo T., Takuma K., Nakahara M., Oura K. (2022). Characterization of Cisplatin Effects in Lenvatinib-resistant Hepatocellular Carcinoma Cells. Anticancer Res..

[168] Oura K., Takuma K., Nakahara M., Tadokoro T., Fujita K., Mimura S., Tani J., Morishita A., Kobara H., Masaki T. (2021). Multimodal treatment involving molecular targeted agents and on-demand transcatheter arterial chemoembolization for advanced hepatocellular carcinoma: A case report and review of the literature. Mol. Clin. Oncol..

[169] Baterdene O., Miura K., Ueno W., Watanabe S., Tsukui M., Nomoto H., Goka R., Maeda H., Yamamoto H., Morimoto N. (2022). A successful case of transarterial chemoembolization for hyperprogressive disease induced by immunotherapy in a patient with unresectable hepatocellular carcinoma. Clin. J. Gastroenterol..

[170] Hasegawa H., Kawakubo E., Kitagawa D., Kishihara F., Funahashi S., Kitamura M. (2020). Combined Modality Therapy for Giant Hepatocellular Carcinoma and Multiple Lung Metastases-A Case Study. Gan Kagaku Ryoho.

[171] Montasser A., Beaufrere A., Cauchy F., Bouattour M., Soubrane O., Albuquerque M., Paradis V. (2021). Transarterial chemoembolisation enhances programmed death-1 and programmed death-ligand 1 expression in hepatocellular carcinoma. Histopathology.

[172] Sun L., Xu X., Meng F., Liu Q., Wang H., Li X., Li G., Chen F. (2022). Lenvatinib plus transarterial chemoembolization with or without immune checkpoint inhibitors for unresectable hepatocellular carcinoma: A review. Front. Oncol..

[173] Fournel L., Wu Z., Stadler N., Damotte D., Lococo F., Boulle G., Segal-Bendirdjian E., Bobbio A., Icard P., Tredaniel J. (2019). Cisplatin increases PD-L1 expression and optimizes immune check-point blockade in non-small cell lung cancer. Cancer Lett..

[174] Tran L., Allen C.T., Xiao R., Moore E., Davis R., Park S.J., Spielbauer K., Van Waes C., Schmitt N.C. (2017). Cisplatin Alters Antitumor Immunity and Synergizes with PD-1/PD-L1 Inhibition in Head and Neck Squamous Cell Carcinoma. Cancer Immunol. Res..

[175] Li S., Ji J., Zhang Z., Peng Q., Hao L., Guo Y., Zhou W., Cui Q., Shi X. (2020). Cisplatin promotes the expression level of PD-L1 in the microenvironment of hepatocellular carcinoma through YAP1. Mol. Cell Biochem..

[176] Zhang Z.S., Yang R.H., Yao X., Cheng Y.Y., Shi H.X., Yao C.Y., Gao Z.X., Qi D.F., Zhang W.K., Dou Y.Y. (2021). HGF/c-MET pathway contributes to cisplatin-mediated PD-L1 expression in hepatocellular carcinoma. Cell Biol. Int..

[177] Li W., Pei Y., Wang Z., Liu J. (2022). Efficacy of transarterial chemoembolization monotherapy or combination conversion therapy in unresectable hepatocellular carcinoma: A systematic review and meta-analysis. Front. Oncol..

[178] Kaibori M., Matsushima H., Ishizaki M., Kosaka H., Matsui K., Nakatani M., Kariya S., Yamaguchi T., Yoshida K., Yoshii K. (2022). The Impact of Sorafenib in Combination with Intermittent Hepatic Arterial Infusion Chemotherapy for Unresectable Hepatocellular Carcinoma with Major Vascular Invasion. Cancer Investig..

[179] Kondo M., Morimoto M., Kobayashi S., Ohkawa S., Hidaka H., Nakazawa T., Aikata H., Hatanaka T., Takizawa D., Matsunaga K. (2019). Randomized, phase II trial of sequential hepatic arterial infusion chemotherapy and sorafenib versus sorafenib alone as initial therapy for advanced hepatocellular carcinoma: SCOOP-2 trial. BMC Cancer.

[180] Choi J.H., Chung W.J., Bae S.H., Song D.S., Song M.J., Kim Y.S., Yim H.J., Jung Y.K., Suh S.J., Park J.Y. (2018). Randomized, prospective, comparative study on the effects and safety of sorafenib vs. hepatic arterial infusion chemotherapy in patients with advanced hepatocellular carcinoma with portal vein tumor thrombosis. Cancer Chemother. Pharm..

[181] Hatooka M., Kawaoka T., Aikata H., Inagaki Y., Morio K., Nakahara T., Murakami E., Tsuge M., Hiramatsu A., Imamura M. (2018). Hepatic arterial infusion chemotherapy followed by sorafenib in patients with advanced hepatocellular carcinoma (HICS 55): An open label, non-comparative, phase II trial. BMC Cancer.

[182] Ishizaki M., Kaibori M., Matsui K., Ikeda H., Yoshida K., Okazaki K., Kariya S., Tanigawa N., Nakatake R., Matsushima H. (2017). Phase I Study of Sorafenib in Combination with Intermittent Hepatic Arterial Infusion Chemotherapy for Unresectable Hepatocellular Carcinoma. Cancer Investig..

[183] Ikeda M., Shimizu S., Sato T., Morimoto M., Kojima Y., Inaba Y., Hagihara A., Kudo M., Nakamori S., Kaneko S. (2016). Sorafenib plus hepatic arterial infusion chemotherapy with cisplatin versus sorafenib for advanced hepatocellular carcinoma: Randomized phase II trial. Ann. Oncol..

[184] Dinh V.Y., Bhatia S., Narayanan G., Yrizarry J., Savaraj N., O’Brien C., Martin P., Feun L. (2016). Pilot Study of Intrahepatic Artery Chemotherapy in Combination with Sorafenib in Hepatocellular Carcinoma. Anticancer Res..

[185] Hagihara A., Ikeda M., Ueno H., Morizane C., Kondo S., Nakachi K., Mitsunaga S., Shimizu S., Kojima Y., Suzuki E. (2014). Phase I study of combination chemotherapy using sorafenib and transcatheter arterial infusion with cisplatin for advanced hepatocellular carcinoma. Cancer Sci..

[186] Schmid I., Haberle B., Albert M.H., Corbacioglu S., Frohlich B., Graf N., Kammer B., Kontny U., Leuschner I., Scheel-Walter H.G. (2012). Sorafenib and cisplatin/doxorubicin (PLADO) in pediatric hepatocellular carcinoma. Pediatr. Blood Cancer.

[187] Zhang L., Gong J.F., Pan H.M., Bai Y.X., Liu T.S., Cheng Y., Chen Y.C., Huang J.Y., Xu T.T., Ge F.J. (2022). Atezolizumab therapy in Chinese patients with locally advanced or metastatic solid tumors: An open-label, phase Ⅰ study. Beijing Da Xue Xue Bao Yi Xue Ban.

[188] Roth G.S., Neuzillet C., Sarabi M., Edeline J., Malka D., Lievre A. (2023). Cholangiocarcinoma: What are the options in all comers and how has the advent of molecular profiling opened the way to personalised medicine ?. Eur. J. Cancer.

[189] Vithayathil M., Khan S.A. (2022). Current epidemiology of cholangiocarcinoma in Western countries. J. Hepatol..

[190] Khan S.A., Thomas H.C., Davidson B.R., Taylor-Robinson S.D. (2005). Cholangiocarcinoma. Lancet.

[191] Mosconi S., Beretta G.D., Labianca R., Zampino M.G., Gatta G., Heinemann V. (2009). Cholangiocarcinoma. Crit. Rev. Oncol. Hematol..

[192] Valle J.W., Wasan H., Johnson P., Jones E., Dixon L., Swindell R., Baka S., Maraveyas A., Corrie P., Falk S. (2009). Gemcitabine alone or in combination with cisplatin in patients with advanced or metastatic cholangiocarcinomas or other biliary tract tumours: A multicentre randomised phase II study—The UK ABC-01 Study. Br. J. Cancer.

[193] Morizane C., Okusaka T., Mizusawa J., Takashima A., Ueno M., Ikeda M., Hamamoto Y., Ishii H., Boku N., Furuse J. (2013). Randomized phase II study of gemcitabine plus S-1 versus S-1 in advanced biliary tract cancer: A Japan Clinical Oncology Group trial (JCOG 0805). Cancer Sci..

[194] Okusaka T., Nakachi K., Fukutomi A., Mizuno N., Ohkawa S., Funakoshi A., Nagino M., Kondo S., Nagaoka S., Funai J. (2010). Gemcitabine alone or in combination with cisplatin in patients with biliary tract cancer: A comparative multicentre study in Japan. Br. J. Cancer.

[195] Kanai M., Hatano E., Kobayashi S., Fujiwara Y., Marubashi S., Miyamoto A., Shiomi H., Kubo S., Ikuta S., Yanagimoto H. (2015). A multi-institution phase II study of gemcitabine/cisplatin/S-1 (GCS) combination chemotherapy for patients with advanced biliary tract cancer (KHBO 1002). Cancer Chemother. Pharm..

[196] Sakai D.M., Zornow K.A., Campoy L., Cable C., Appel L.D., Putnam H.J., Martin-Flores M. (2018). Intravenous rocuronium 0.3 mg/kg improves the conditions for tracheal intubation in cats: A randomized, placebo-controlled trial. J. Feline Med. Surg..

[197] Banales J.M., Marin J.J.G., Lamarca A., Rodrigues P.M., Khan S.A., Roberts L.R., Cardinale V., Carpino G., Andersen J.B., Braconi C. (2020). Cholangiocarcinoma 2020: The next horizon in mechanisms and management. Nat. Rev. Gastroenterol. Hepatol..

[198] Valle J.W., Wasan H., Lopes A., Backen A.C., Palmer D.H., Morris K., Duggan M., Cunningham D., Anthoney D.A., Corrie P. (2015). Cediranib or placebo in combination with cisplatin and gemcitabine chemotherapy for patients with advanced biliary tract cancer (ABC-03): A randomised phase 2 trial. Lancet Oncol..

[199] Oh D.Y., Lee K.H., Lee D.W., Yoon J., Kim T.Y., Bang J.H., Nam A.R., Oh K.S., Kim J.M., Lee Y. (2022). Gemcitabine and cisplatin plus durvalumab with or without tremelimumab in chemotherapy-naive patients with advanced biliary tract cancer: An open-label, single-centre, phase 2 study. Lancet Gastroenterol. Hepatol..

[200] Zheng H.C. (2017). The molecular mechanisms of chemoresistance in cancers. Oncotarget.

[201] Kiss R.C., Xia F., Acklin S. (2021). Targeting DNA Damage Response and Repair to Enhance Therapeutic Index in Cisplatin-Based Cancer Treatment. Int. J. Mol. Sci..

[202] Kawaguchi H., Terai Y., Tanabe A., Sasaki H., Takai M., Fujiwara S., Ashihara K., Tanaka Y., Tanaka T., Tsunetoh S. (2014). Gemcitabine as a molecular targeting agent that blocks the Akt cascade in platinum-resistant ovarian cancer. J. Ovarian Res..

[203] Yang Q., Nie Y.H., Cai M.B., Li Z.M., Zhu H.B., Tan Y.R. (2022). Gemcitabine Combined with Cisplatin Has a Better Effect in the Treatment of Recurrent/Metastatic Advanced Nasopharyngeal Carcinoma. Drug Des. Devel Ther..

[204] Zhang Y., Chen L., Hu G.Q., Zhang N., Zhu X.D., Yang K.Y., Jin F., Shi M., Chen Y.P., Hu W.H. (2019). Gemcitabine and Cisplatin Induction Chemotherapy in Nasopharyngeal Carcinoma. N. Engl. J. Med..

[205] Ding D.Y., Gan X.J., Zhang J.N., Hou G.J., Tao Q.F., Sun D.P., Li W., Yang Y., Ding W.B., Yu J. (2023). Serum thrombospondin-1 serves as a novel biomarker and agonist of gemcitabine-based chemotherapy in intrahepatic cholangiocarcinoma. Br. J. Cancer.

[206] Prasopporn S., Suppramote O., Ponvilawan B., Jamyuang C., Chanthercrob J., Chaiboonchoe A., More-Krong P., Kongsri K., Suntiparpluacha M., Chanwat R. (2022). Combining the SMAC mimetic LCL161 with Gemcitabine plus Cisplatin therapy inhibits and prevents the emergence of multidrug resistance in cholangiocarcinoma. Front. Oncol..

[207] Huang C.S., Zhu Y.Q., Xu Q.C., Chen S., Huang Y., Zhao G., Ni X., Liu B., Zhao W., Yin X.Y. (2022). YTHDF2 promotes intrahepatic cholangiocarcinoma progression and desensitises cisplatin treatment by increasing CDKN1B mRNA degradation. Clin. Transl. Med..

[208] Schmid I., von Schweinitz D. (2017). Pediatric hepatocellular carcinoma: Challenges and solutions. J. Hepatocell. Carcinoma.

[209] Hager J., Sergi C.M., Sergi C.M. (2021). Hepatoblastoma. Liver Cancer.

[210] Turcotte L.M., Georgieff M.K., Ross J.A., Feusner J.H., Tomlinson G.E., Malogolowkin M.H., Krailo M.D., Miller N., Fonstad R., Spector L.G. (2014). Neonatal medical exposures and characteristics of low birth weight hepatoblastoma cases: A report from the Children’s Oncology Group. Pediatr. Blood Cancer.

[211] Digiacomo G., Serra R.P., Turrini E., Tiri A., Cavazzoni A., Alfieri R., Bertolini P. (2023). State of the art and perspectives in pediatric hepatocellular carcinoma. Biochem. Pharm..

[212] Khanna R., Verma S.K. (2018). Pediatric hepatocellular carcinoma. World J. Gastroenterol..

[213] Chan K.L., Fan S.T., Tam P.K., Chiang A.K., Chan G.C., Ha S.Y. (2002). Paediatric hepatoblastoma and hepatocellular carcinoma: Retrospective study. Hong Kong Med. J..

[214] Pritchard J., Brown J., Shafford E., Perilongo G., Brock P., Dicks-Mireaux C., Keeling J., Phillips A., Vos A., Plaschkes J. (2000). Cisplatin, doxorubicin, and delayed surgery for childhood hepatoblastoma: A successful approach--results of the first prospective study of the International Society of Pediatric Oncology. J. Clin. Oncol..

[215] Czauderna P., Mackinlay G., Perilongo G., Brown J., Shafford E., Aronson D., Pritchard J., Chapchap P., Keeling J., Plaschkes J. (2002). Hepatocellular carcinoma in children: Results of the first prospective study of the International Society of Pediatric Oncology group. J. Clin. Oncol..

[216] Czauderna P. (2002). Adult type vs. Childhood hepatocellular carcinoma--are they the same or different lesions? Biology, natural history, prognosis, and treatment. Med. Pediatr. Oncol..

[217] Perilongo G., Shafford E., Maibach R., Aronson D., Brugieres L., Brock P., Childs M., Czauderna P., MacKinlay G., Otte J.B. (2004). Risk-adapted treatment for childhood hepatoblastoma. final report of the second study of the International Society of Paediatric Oncology--SIOPEL 2. Eur. J. Cancer.

[218] Czauderna P. (2012). Hepatoblastoma throughout SIOPEL trials—Clinical lessons learnt. Front. Biosci. (Elite Ed).

[219] Murawski M., Weeda V.B., Maibach R., Morland B., Roebuck D.J., Zimmerman A., Casanova M., Perilongo G., Laithier V., Kebudi R. (2016). Hepatocellular Carcinoma in Children: Does Modified Platinum- and Doxorubicin-Based Chemotherapy Increase Tumor Resectability and Change Outcome? Lessons Learned From the SIOPEL 2 and 3 Studies. J. Clin. Oncol..

[220] Czauderna P., Haeberle B., Hiyama E., Rangaswami A., Krailo M., Maibach R., Rinaldi E., Feng Y., Aronson D., Malogolowkin M. (2016). The Children’s Hepatic tumors International Collaboration (CHIC): Novel global rare tumor database yields new prognostic factors in hepatoblastoma and becomes a research model. Eur. J. Cancer.

[221] Meyers R.L., Maibach R., Hiyama E., Haberle B., Krailo M., Rangaswami A., Aronson D.C., Malogolowkin M.H., Perilongo G., von Schweinitz D. (2017). Risk-stratified staging in paediatric hepatoblastoma: A unified analysis from the Children’s Hepatic tumors International Collaboration. Lancet Oncol..

[222] Knight K.R., Kraemer D.F., Neuwelt E.A. (2005). Ototoxicity in children receiving platinum chemotherapy: Underestimating a commonly occurring toxicity that may influence academic and social development. J. Clin. Oncol..

[223] Bass J.K., Knight K.R., Yock T.I., Chang K.W., Cipkala D., Grewal S.S. (2016). Evaluation and Management of Hearing Loss in Survivors of Childhood and Adolescent Cancers: A Report From the Children’s Oncology Group. Pediatr. Blood Cancer.

[224] Romano A., Capozza M.A., Mastrangelo S., Maurizi P., Triarico S., Rolesi R., Attina G., Fetoni A.R., Ruggiero A. (2020). Assessment and Management of Platinum-Related Ototoxicity in Children Treated for Cancer. Cancers.

[225] Grewal S., Merchant T., Reymond R., McInerney M., Hodge C., Shearer P. (2010). Auditory late effects of childhood cancer therapy: A report from the Children’s Oncology Group. Pediatrics.

[226] Clemens E., van den Heuvel-Eibrink M.M., Mulder R.L., Kremer L.C.M., Hudson M.M., Skinner R., Constine L.S., Bass J.K., Kuehni C.E., Langer T. (2019). Recommendations for ototoxicity surveillance for childhood, adolescent, and young adult cancer survivors: A report from the International Late Effects of Childhood Cancer Guideline Harmonization Group in collaboration with the PanCare Consortium. Lancet Oncol..

[227] Brock P.R., Maibach R., Neuwelt E.A. (2018). Sodium Thiosulfate and Cisplatin-Induced Hearing Loss. N. Engl. J. Med..

[228] Freyer D.R., Brock P.R., Chang K.W., Dupuis L.L., Epelman S., Knight K., Mills D., Phillips R., Potter E., Risby D. (2020). Prevention of cisplatin-induced ototoxicity in children and adolescents with cancer: A clinical practice guideline. Lancet Child Adolesc. Health.

[229] Neuwelt E.A., Gilmer-Knight K., Lacy C., Nicholson H.S., Kraemer D.F., Doolittle N.D., Hornig G.W., Muldoon L.L. (2006). Toxicity profile of delayed high dose sodium thiosulfate in children treated with carboplatin in conjunction with blood-brain-barrier disruption. Pediatr. Blood Cancer.

[230] Fouladi M., Chintagumpala M., Ashley D., Kellie S., Gururangan S., Hassall T., Gronewold L., Stewart C.F., Wallace D., Broniscer A. (2008). Amifostine protects against cisplatin-induced ototoxicity in children with average-risk medulloblastoma. J. Clin. Oncol..

[231] Campbell K.C., Meech R.P., Klemens J.J., Gerberi M.T., Dyrstad S.S., Larsen D.L., Mitchell D.L., El-Azizi M., Verhulst S.J., Hughes L.F. (2007). Prevention of noise- and drug-induced hearing loss with D-methionine. Hear. Res..

